# Supplementary material for: Forward-predictive SERS-based chemical taxonomy for untargeted structural elucidation of epimeric cerebrosides
Source: Nat Commun. 2024 Mar 22;15:2582. doi: 10.1038/s41467-024-46838-z (PMC10960001; doi:10.1038/s41467-024-46838-z)
Supplement: Supplementary file 1 — Supplementary Information [file 41467_2024_46838_MOESM1_ESM.pdf]

Supplementary Information

Forward-predictive SERS-based chemical taxonomy for untargeted structural elucidation of epimeric cerebrosides

Emily Xi Tan[1], Shi Xuan Leong [1], Wei An Liew[1], In Yee Phang,[2] Jie Ying Ng[3], Nguan Soon Tan[4,5] , Yie Hou Lee[3,6,7*], Xing Yi Ling[1,2,4,8*]

Affiliations:

[1] School of Chemistry, Chemical Engineering and Biotechnology, Nanyang Technological University, 21 Nanyang Link, Singapore 637371.

[2] School of Chemical and Material Engineering, Jiangnan University, Wuxi, 214122, People's Republic of China.

[3] KK Research Centre, KKH, 100 Bukit Timah Road Singapore 229899

[4] Lee Kong Chian School of Medicine, Nanyang Technological University, 59 Nanyang Drive, Singapore 636921.

[5] School of Biological Sciences, Nanyang Technological University Singapore, 60 Nanyang Drive, 637551 Singapore, Singapore

[6] Obstetrics and Gynaecology Academic Clinical Program, Duke-NUS Medical School, Singapore 169857, Singapore

[7] Critical Analytics in Manufacturing Personalized Medicine, Singapore-MIT Alliance for Research and Technology, 1 CREATE Way, #04-13/14 Enterprise Wing, Singapore 138602.

[8] Institute for Digital Molecular Analytics and Science (IDMxS), Nanyang Technological University, 59 Nanyang Drive, Singapore 636921.

* Correspondence to: yiehou.lee@smart.mit.edu; xyling@ntu.edu.sg

Supplementary Note 1. Characterization of silver nanocubes (Ag NCs).

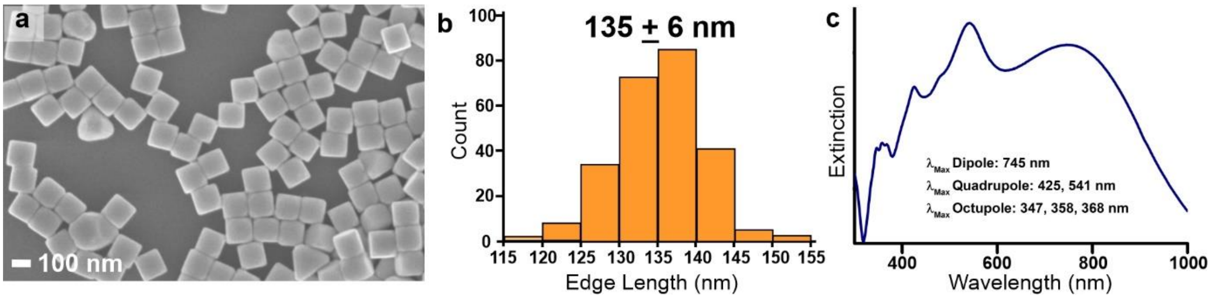

Supplementary Figure 1. Ag nanocube characterization. a. SEM image, **b.** Edge length distribution, and **c.** Normalized UV-vis extinction spectrum of Ag nanocubes.

Supplementary Note 2. SERS platform signal intensity and reproducibility study.

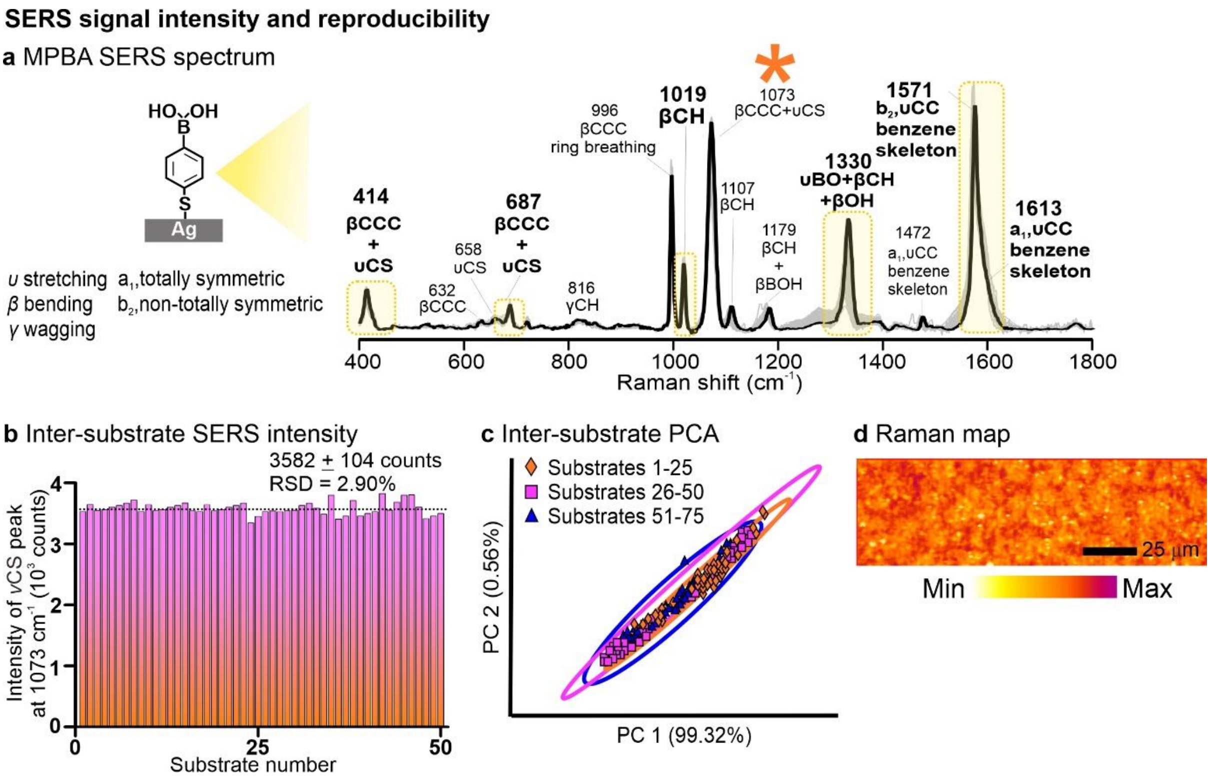

Supplementary Figure 2. SERS signal intensity and reproducibility. a. SERS spectra of MPBA with C-S stretching (a_1, βCCC + νCS) peak at 1073 cm^{-1} highlighted. **b.** Inter-substrate SERS intensity at 1073 cm^{-1} was calculated over 50 individual substrates with a low relative standard deviation of 2.90%. **c.** Inter-substrate principal component analysis (PCA) of 75 randomly selected substrates over 3 different batches of functionalized 4-MPBA-Ag nanocubes showing full overlaps of 95% confidence ellipses, demonstrating high spectral reproducibility. d. SERS hyperspectra of 4-MPBA-Ag nanocubes over an area of 50 ×150 μm^2 showing high signal homogeneity.

Supplementary Note 3. Hydrophobic Ag film SERS platform characterization.

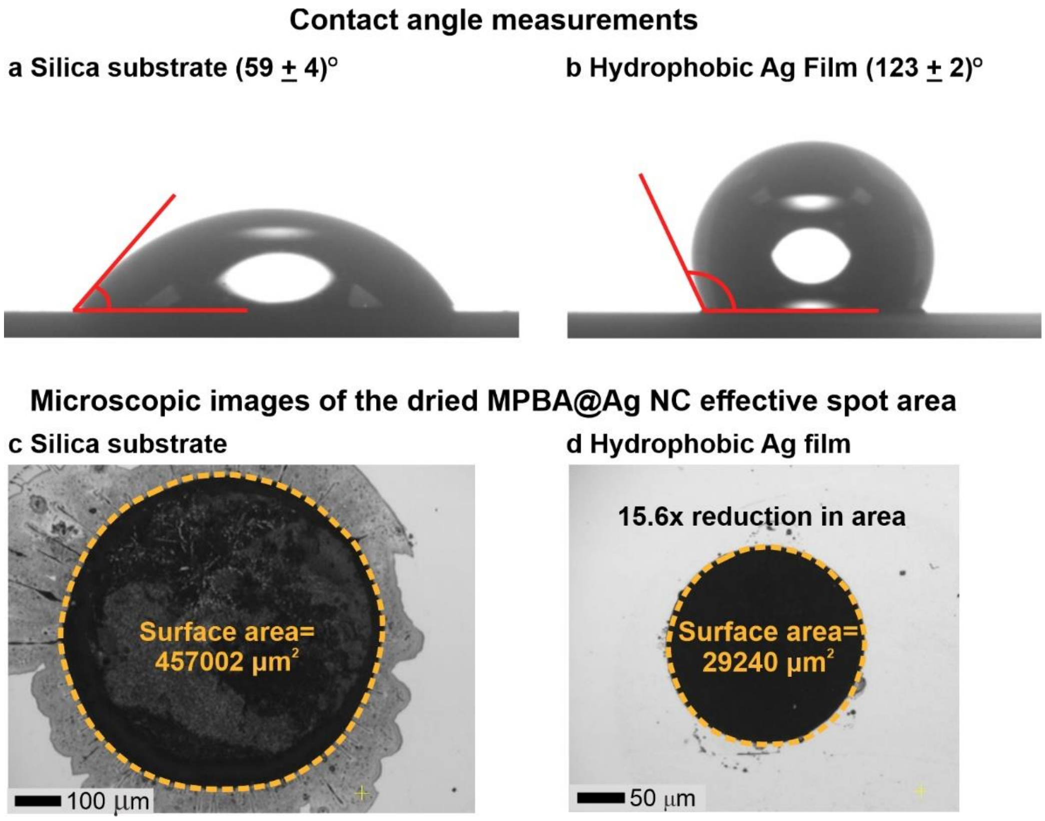

Supplementary Figure 3. Hydrophobic Ag film SERS substrate characterization. (A) Contact angle measurement and (B) Microscopic images of the dried 2 μL of 4-MPBA-Ag nanocubes showing the physical concentrating effect of and a 15.6 times reduction in effective spot area on the hydrophobic substrate for SERS measurements.

Supplementary Note 4. Determining the analytical enhancement factor (AEF).

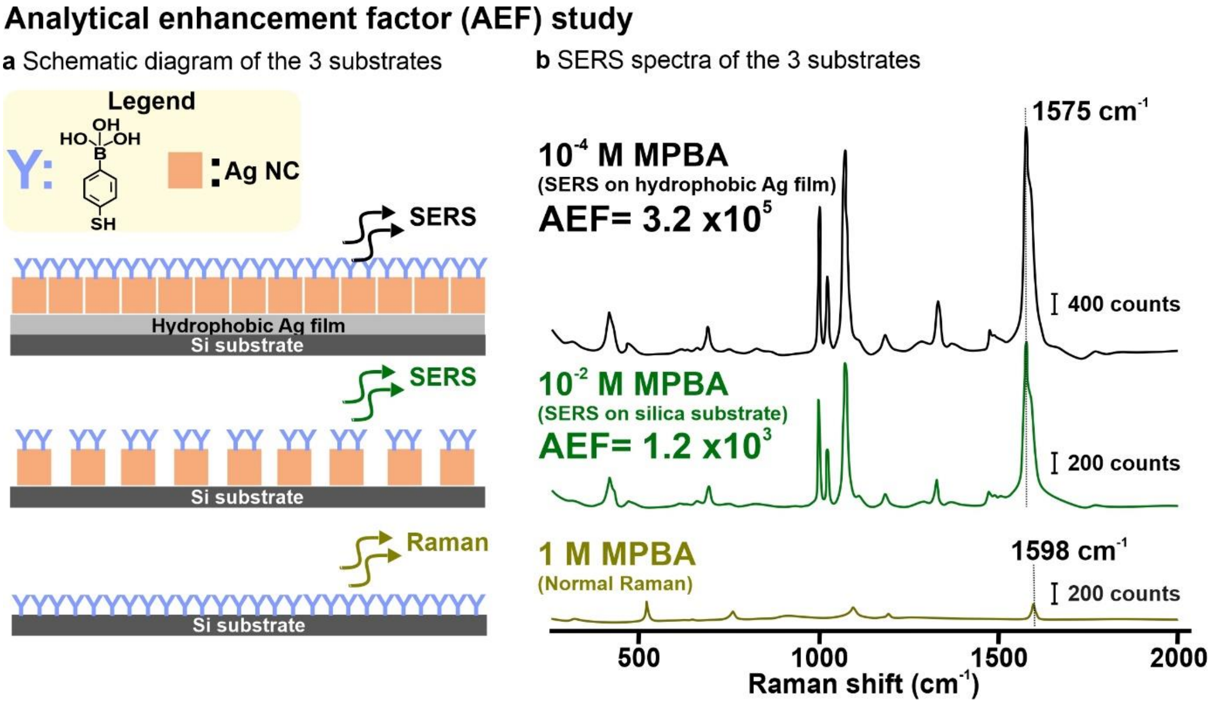

Supplementary Figure 4. Analytical enhancement factor study conducted. (A) Schematic of the 4-MPBA@Si substrate used for normal Raman of 4-MPBA, the 4-MPBA-Ag@Si substrate used for SERS measurement of 4-MPBA, and 4-MPBA-Ag@Hydrophobic Ag film substrate used for SERS measurement of 4-MPBA. (B) Normal Raman spectrum and SERS spectra of 4-MPBA on the three different substrates, with the hydrophobic substrate yielding the highest AEF of 3.2 x 10^5.

Supplementary Note 5. Principal component analysis (PCA) and bond angle comparison between GlcCer and GalCer adducts.

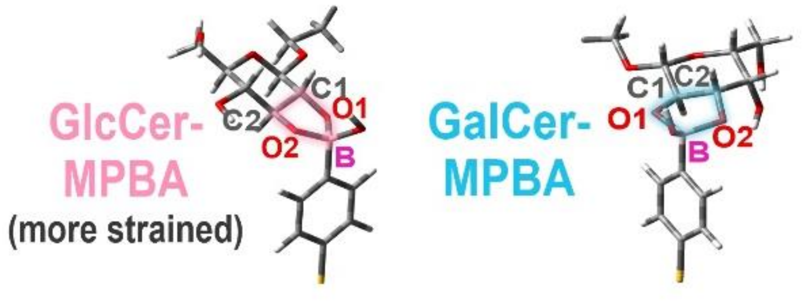

Atom	Bond angle (°)	
	GlcCer	GlaCer
B	104.5	106.2
O_1	105.1	103.2
O_2	105.4	106.2
C_1	102.7	102.8
C_2	102.7	102.8

Supplementary Figure 5. Representative bond angle comparison of GlcCer-MPBA and GalCer-MPBA adducts in optimized calculated configurations.

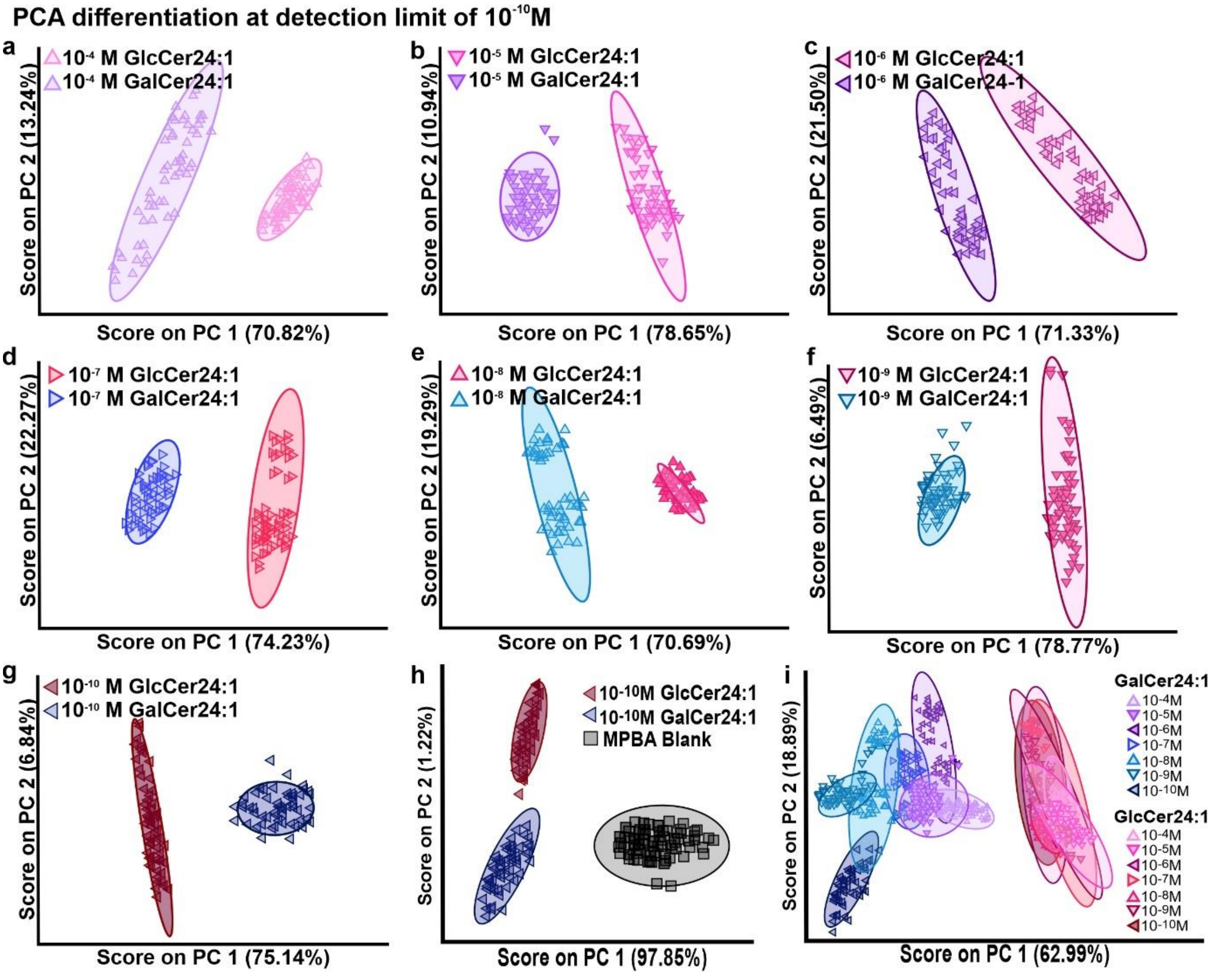

Supplementary Figure 6. PCA differentiation of epimers GlcCer24:1 and GalCer24:1 from 10^{-4} M to 10^{-10} M. All PCA plots show clear separation at every concentration.

In the PCA of epimeric $GlcCer_{24:1}$-MPBA, and $GalCer_{24:1}$-MPBA at different concentrations ranging from 10^{-4} M to 10^{-10} M, we observe two distinct clusters of the two along PC1 for all test instances, indicating high consistency and specificity of our strategy. Amongst the blank MPBA, $GlcCer_{24:1}$-MPBA, and $GalCer_{24:1}$-MPBA, we observe the clear separation of the blank from analytes along the major PC1 axis and further separation of the two analytes along PC2, confirming ultra-trace sensitivity and the detection limit of 10^{-10} M.

Supplementary Note 6. Feature engineering.

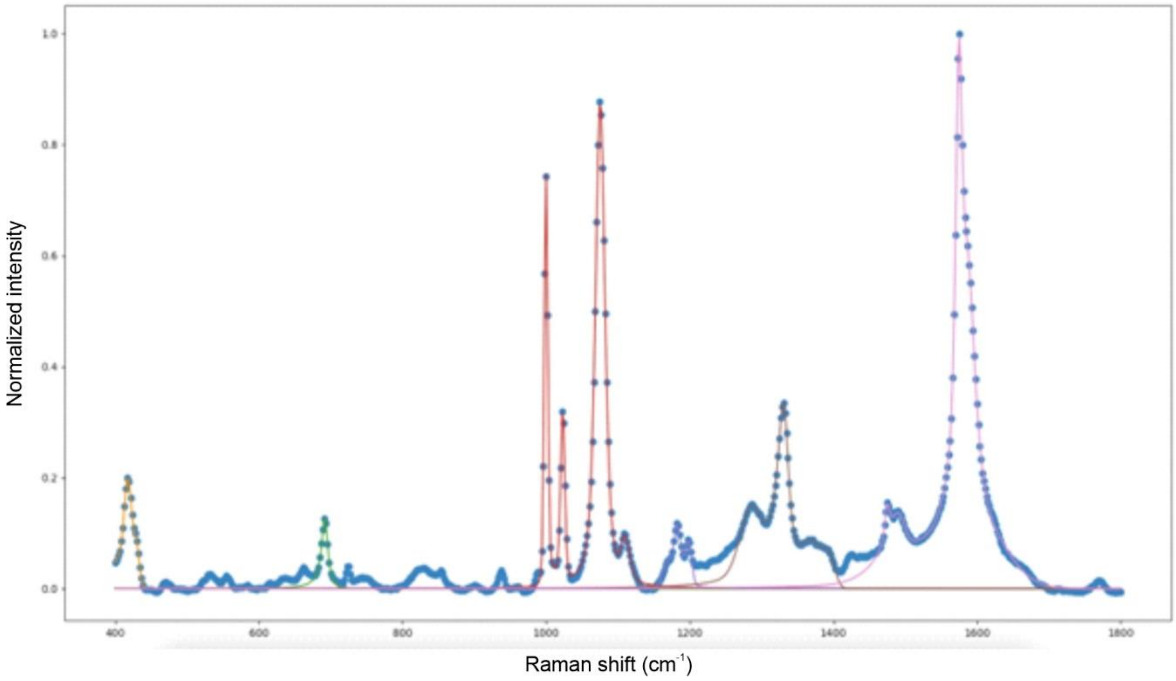

Supplementary Figure 7. Automated spectra deconvolution of the SERS spectra using a pseudo-Voigt profile.

To deconvolute the SERS spectra and resolve the overlapping peaks, we first divide all normalized and baselined SERS spectra into 6 regions and then fit the peaks within each region with pseudo-Voigt profiles, using an automated program. The initial value of the peak position inputted will provide a “first guess” for the fitting, however, it does not dictate the final position as the iterative process will test different combinations of pseudo-Voigt peaks and only stop when a minimal error (threshold=1 Difference (%) between cumulative fitted spectra and original spectra) is reached. Regions 1, 2, 3, 4, 5, and 6 are fitted with 2, 2, 4, 3, 4, and 4 peaks respectively, totaling 19 peaks. For each peak, 5 parameters are generated, namely, peak position, intensity, full width at half maximum (FWHM), skew (denotes any peak trailing, max = 1 (skew left), min = -1 (skew right), perfectly symmetrical peaks = 0) and a ratio (percentage composition of Lorentzian vs Gaussian components in each peak, where 1 = fully Lorentzian, 0 = fully Gaussian). The final output will yield 19 × 5 = 95 components/parameters that are discrete and structured to leverage machine learning models.

Supplementary Table 1. Example of the 95 spectral features extracted from the SERS spectra of $GalCer_8$.

Peak	Spectral feature	$GalCer_8$
**1**	Fraction	0.370667
	Skew	-1.25427
	Position (cm^{-1})	416.8806
	Intensity (arb. u)	0.15473
	FWHM	10.6694
**2**	Fraction	0.999988
	Skew	-2.39708
	Position (cm^{-1})	426.657
	Intensity (arb. u)	0.093737
	FWHM	14.92072
**3**	Fraction	0.760682
	Skew	-0.38407
	Position (cm^{-1})	691.8685
	Intensity (arb. u)	0.14005
	FWHM	8.732675
**4**	Fraction	0.294901
	Skew	0.167828
	Position (cm^{-1})	999.8103
	Intensity (arb. u)	0.944911
	FWHM	5.442488
**5**	Fraction	0.444604
	Skew	-0.07943
	Position (cm^{-1})	1022.854
	Intensity (arb. u)	0.408156
	FWHM	6.916914
**6**	Fraction	0.645051
	Skew	0.009794
	Position (cm^{-1})	1073.886
	Intensity (arb. u)	0.826549
	FWHM	11.5775
**7**	Fraction	3.00E-08
	Skew	2.075494
	Position (cm^{-1})	1110.478
	Intensity (arb. u)	0.069999
	FWHM	18.9268
**8**	Fraction	0.060629
	Skew	4.043513
	Position (cm^{-1})	1186.164
	Intensity (arb. u)	0.038516
	FWHM	18.80968
**9**	Fraction	0.000554
	Skew	-0.17562
	Position (cm^{-1})	1182.323
	Intensity (arb. u)	0.060502
	FWHM	8.281648
**10**	Fraction	0.760631
	Skew	2.67135
	Position (cm^{-1})	1289.623
	Intensity (arb. u)	0.107628
	FWHM	26.69685
**11**	Fraction	0.877468
	Skew	-0.30626
	Position (cm^{-1})	1327.777

	Intensity (arb. u)	0.031229
	FWHM	19.1152
**12**	Fraction	0.993306
	Skew	1.370064
	Position (cm^{-1})	1398.442
	Intensity (arb. u)	0.06833
	FWHM	0
**13**	Fraction	0.673183
	Skew	-4.79212
	Position (cm^{-1})	1363.635
	Intensity (arb. u)	0.048693
	FWHM	57.74752
**14**	Fraction	0.999959
	Skew	-0.27595
	Position (cm^{-1})	1474.515
	Intensity (arb. u)	0.084945
	FWHM	5.371958
**15**	Fraction	0.277375
	Skew	-7.52936
	Position (cm^{-1})	1469.383
	Intensity (arb. u)	0.075972
	FWHM	0
**16**	Fraction	1.83E-05
	Skew	-0.49978
	Position (cm^{-1})	1494.486
	Intensity (arb. u)	0.092723
	FWHM	34.69052
**17**	Fraction	0.891648
	Skew	0.225971
	Position (cm^{-1})	1574.929
	Intensity (arb. u)	0.828236
	FWHM	9.528314
**18**	Fraction	2.90E-07
	Skew	7.220597
	Position (cm^{-1})	1619.817
	Intensity (arb. u)	0.066011
	FWHM	48.24519
**19**	Fraction	0.006824
	Skew	-4.74579
	Position (cm^{-1})	1588.368
	Intensity (arb. u)	0.195589
	FWHM	49.85357

Supplementary Note 7. Machine learning exploration and results

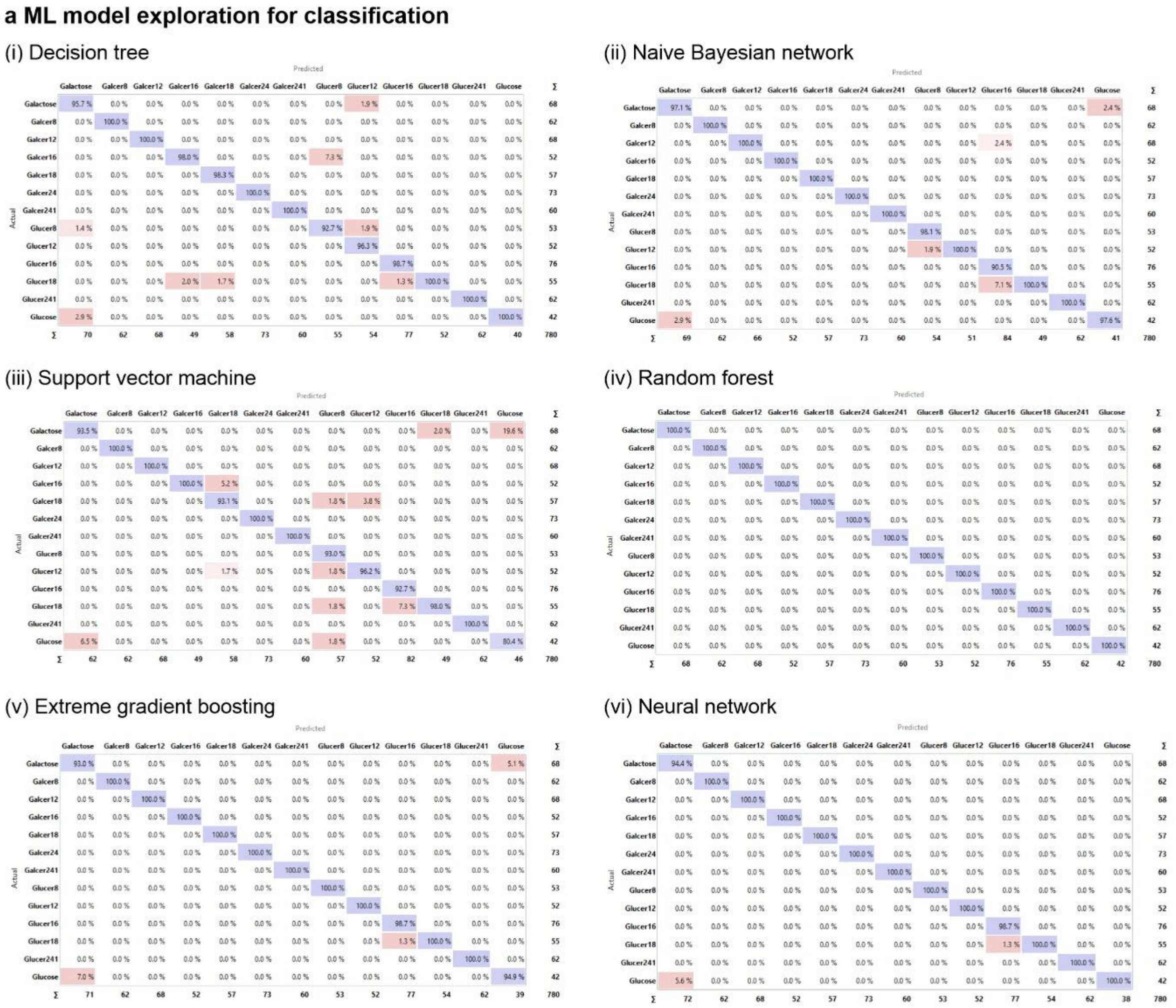

b ML model classification results

Model	Classification accuracy	F1 score	Precision	Recall
Decision tree	0.985	0.985	0.985	0.985
Naive bayesian network	0.985	0.985	0.986	0.985
Support vector machine	0.962	0.961	0.963	0.962
Random forest	**1.000**	1.000	1.000	1.000
Extreme gradient boosting	**0.990**	0.990	0.990	0.990
Neural network	**0.994**	0.994	0.994	0.994

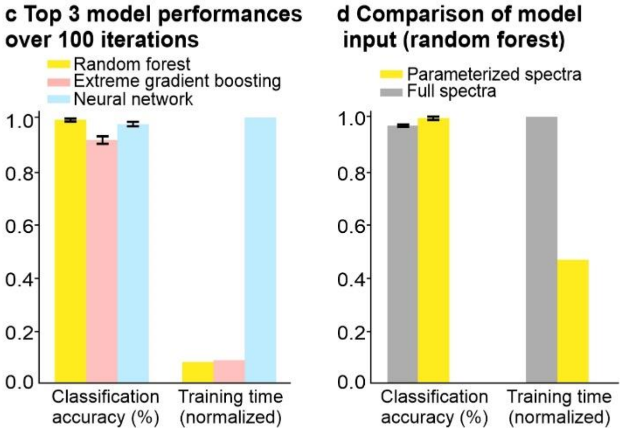

Supplementary Figure 8. Comparison of different ML models for classification of all 13 epimeric biomolecules. a. confusion matrices of (i) decision tree, (ii) naïve Bayesian network, (iii) support vector machine (iv) random forest (v) extreme gradient boosting, and (vi) neural network ML models. **b**. Compiled classification results. **c**. comparison of the top 3 models'

performance over 100 iterations. **d.** Comparison of model inputs used for the best-performing random forest model over 100 iterations.

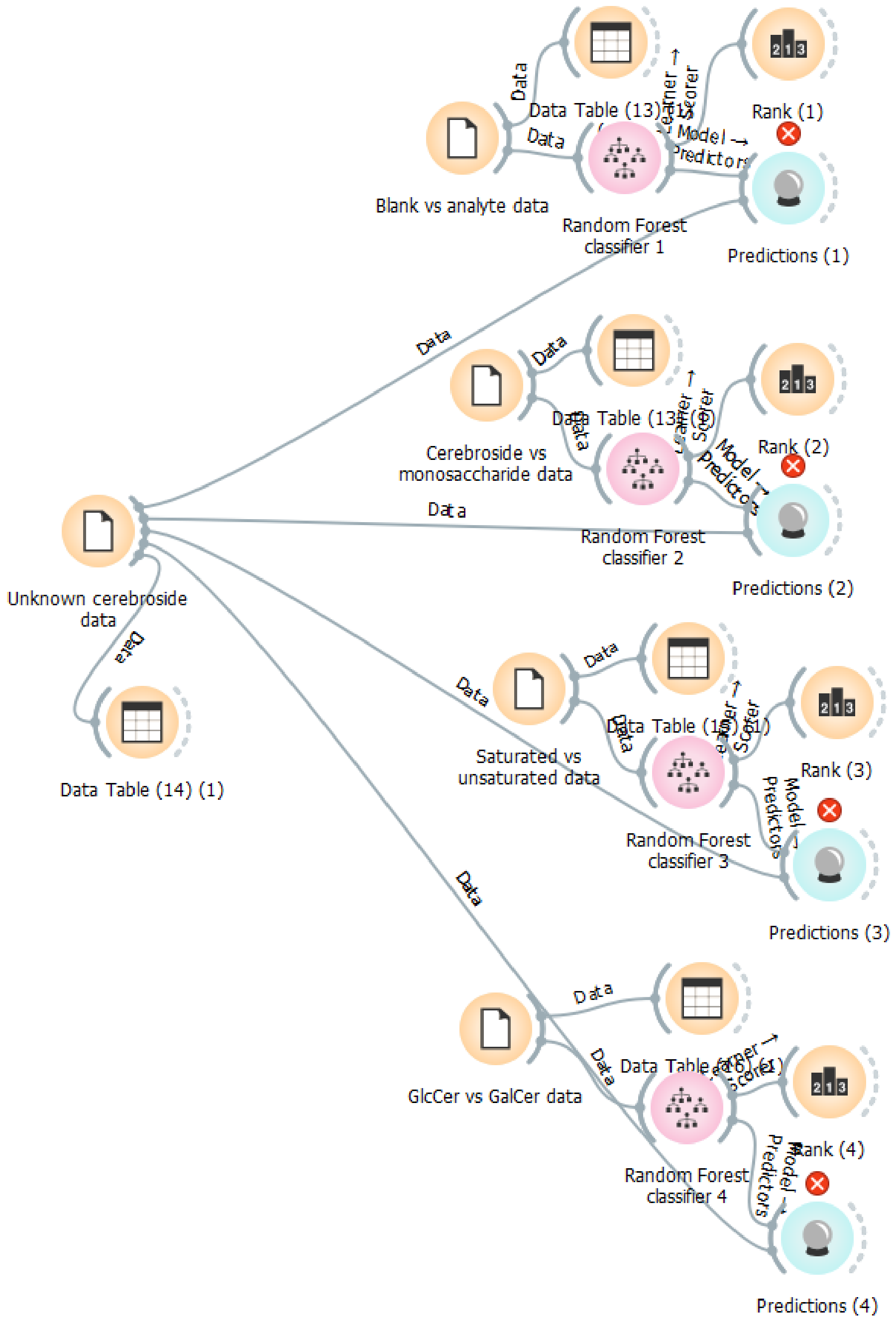

Supplementary Figure 9. Schematics of the forward predictive ML framework. The SERS spectra of unknown cerebrosides, which are untrained in the various ML models within the framework, are hierarchically parsed through the various levels to forward predict the molecular structure. The first random forest classifiers will each identify a specific structural characteristic of the unknown cerebroside based on the input spectra before the spectra are parsed into the support vector machine regressor to determine the carbon chain length.

Forward prediction results of cerebroside carbon chain length. Machine learning regression results for 9 cerebrosides with varying carbon chain lengths at 10^{-4} M. GlcCer test samples are in red and GalCer test samples are in blue, while all training data are indicated in grey. We attribute this figure to Orange, Data Mining Fruitful & Fun (https://orange.biolab.si/.) Orange documentation, content on its website, and other non-code content are all available under Creative Commons Attribution-ShareAlike license unless specified otherwise.

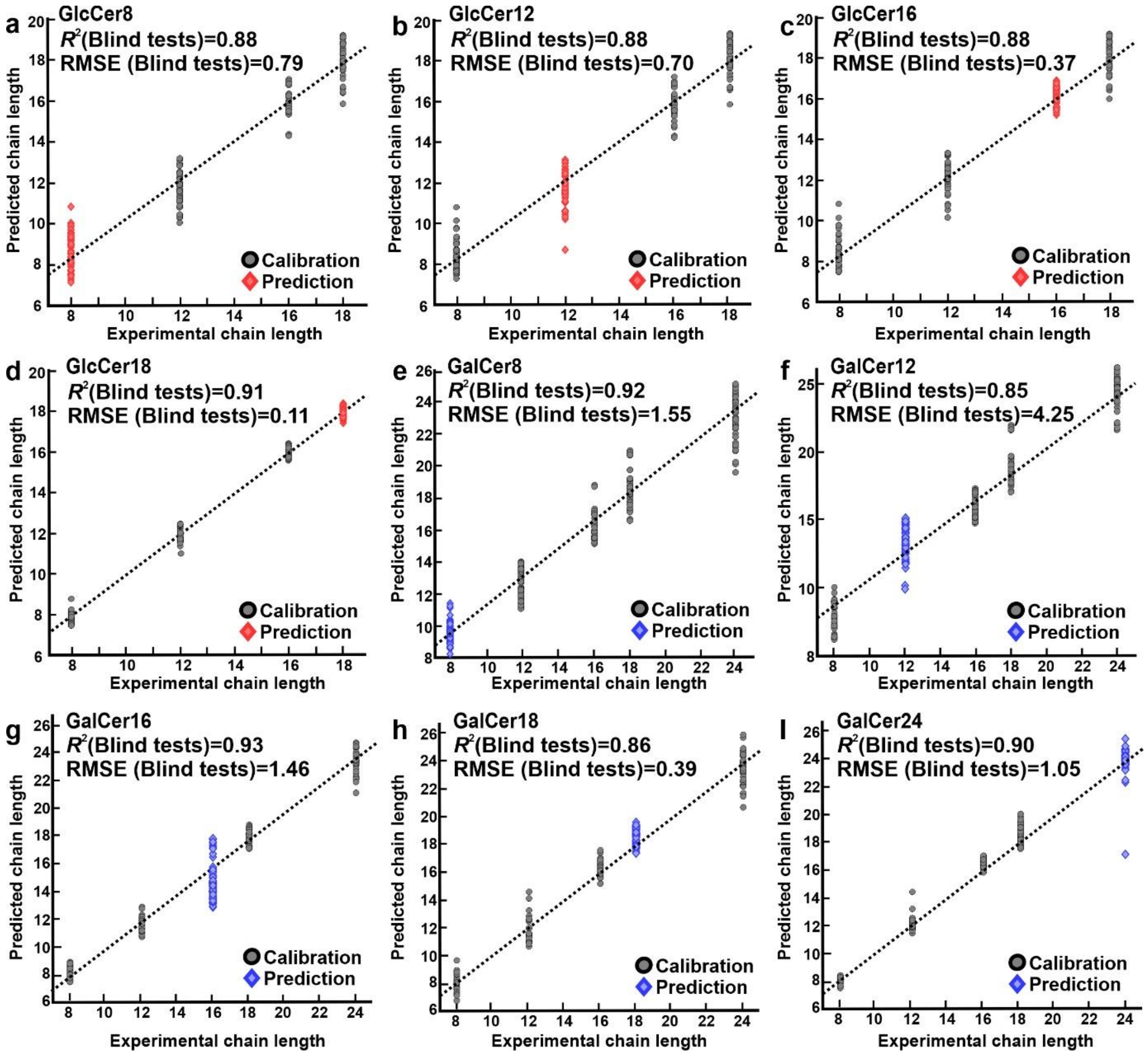

Supplementary Figure 10. Forward prediction regression results. Regression results with the linear best-fit line, R^2 values, and calculated root mean square of prediction (RMSEP) for 9 cerebrosides with varying carbon chain length at 10^{-4} M. The prediction set (blind test) is excluded in model training and only used for model testing and the regression results based on the test set are used to calculate the RMSEP.

Supplementary Table 2. Summary of predicted probability for individual classes and overall classification accuracy of 4 classification models and predicted absolute values (chain length) for the regression model using peak parameters extracted from 60 SERS spectra from 60 $GlcCer_8$ blind test samples. Errors in classification are highlighted in red.

**$GlcCer_8$**										
ML model	Classification model 1		Classification model 2		Classification model 3		Classification model 4		Regression model 5	
Sample no.	Loaded	Blank	Cerebroside	Monosaccharide	Saturated	Unsaturated	GlcCer	GalCer	Predicted chain length	Difference (%)
1	1	0	0.87	0.13	1	0	0.56	0.44	7.99	0.08
2	1	0	0.87	0.13	1	0	0.54	0.46	9.03	12.85
3	1	0	0.95	0.05	1	0	0.49	0.51*	8.96	11.99
4	1	0	0.92	0.08	1	0	0.51	0.49	8.10	1.25
5	1	0	0.92	0.08	1	0	0.56	0.44	9.08	13.49
6	1	0	0.93	0.07	1	0	0.61	0.39	7.90	1.25
7	1	0	0.78	0.22	1	0	0.78	0.22	8.57	7.07
8	1	0	0.9	0.1	1	0	0.52	0.48	8.10	1.25
9	1	0	0.9	0.1	1	0	0.6	0.4	7.90	1.26
10	1	0	0.92	0.08	1	0	0.61	0.39	7.43	7.16
11	1	0	0.91	0.09	1	0	0.53	0.47	8.10	1.25
12	1	0	0.92	0.08	1	0	0.54	0.46	8.76	9.55
13	1	0	0.85	0.15	1	0	0.56	0.44	7.90	1.25
14	1	0	0.95	0.05	1	0	0.95	0.05	8.10	1.25
15	1	0	0.96	0.04	1	0	0.53	0.47	8.10	1.25
16	1	0	0.77	0.23	0.99	0.01	0.57	0.43	7.98	0.31
17	1	0	0.75	0.25	1	0	0.75	0.25	7.54	5.70
18	1	0	0.8	0.2	1	0	0.55	0.45	8.10	1.25
19	1	0	0.76	0.24	1	0	0.54	0.46	8.10	1.25
20	1	0	0.79	0.21	1	0	0.53	0.47	8.21	2.61
21	1	0	0.71	0.29	1	0	0.47	0.53*	8.88	11.02
22	1	0	0.65	0.35	0.99	0.01	0.56	0.44	8.42	5.21
23	1	0	0.69	0.31	1	0	0.55	0.45	7.90	1.24
24	1	0	0.64	0.36	1	0	0.42	0.58*	8.15	1.82
25	1	0	0.8	0.2	1	0	0.55	0.45	7.65	4.35
26	1	0	0.83	0.17	1	0	0.59	0.41	7.90	1.25
27	1	0	0.78	0.22	1	0	0.51	0.49	7.46	6.78
28	1	0	0.77	0.23	1	0	0.54	0.46	7.90	1.24
29	1	0	0.84	0.16	0.99	0.01	0.52	0.48	8.05	0.68
30	1	0	0.81	0.19	1	0	0.55	0.45	8.53	6.57
31	1	0	0.83	0.17	1	0	0.64	0.36	8.10	1.25
32	1	0	0.86	0.14	1	0	0.6	0.4	9.43	17.90
33	1	0	0.94	0.06	1	0	0.94	0.06	10.13	26.65
34	1	0	0.91	0.09	1	0	0.56	0.44	8.29	3.64
35	1	0	0.91	0.09	1	0	0.57	0.43	7.45	6.91
36	1	0	0.91	0.09	1	0	0.53	0.47	7.86	1.71
37	1	0	0.73	0.27	1	0	0.53	0.47	8.10	1.25
38	1	0	0.74	0.26	1	0	0.62	0.38	8.73	9.15
39	1	0	0.74	0.26	1	0	0.68	0.32	8.30	3.76
40	1	0	0.96	0.04	1	0	0.56	0.44	8.10	1.25
41	1	0	0.95	0.05	0.97	0.03	0.54	0.46	9.11	13.93
42	1	0	0.93	0.07	1	0	0.63	0.37	8.10	1.25
43	1	0	0.95	0.05	1	0	0.57	0.43	9.40	17.54
44	1	0	0.92	0.08	0.99	0.01	0.62	0.38	7.77	2.85
45	1	0	0.91	0.09	1	0	0.6	0.4	9.29	16.15

46	1	0	0.83	0.17	1	0	0.68	0.32	9.75	21.82
47	1	0	0.89	0.11	1	0	0.63	0.37	8.74	9.21
48	1	0	0.95	0.05	1	0	0.53	0.47	8.10	1.25
49	1	0	0.96	0.04	1	0	0.53	0.47	7.90	1.25
50	1	0	0.96	0.04	1	0	0.57	0.43	8.39	4.82
51	1	0	0.94	0.06	1	0	0.62	0.38	9.84	23.00
52	1	0	0.81	0.19	1	0	0.62	0.38	8.10	1.25
53	1	0	0.89	0.11	1	0	0.59	0.41	8.91	11.38
54	1	0	0.87	0.13	1	0	0.67	0.33	8.23	2.88
55	1	0	0.94	0.06	1	0	0.59	0.41	7.28	9.01
56	1	0	0.95	0.05	1	0	0.63	0.37	8.98	12.29
57	1	0	0.94	0.06	1	0	0.59	0.41	10.82	35.19
58	1	0	0.94	0.06	0.99	0.01	0.66	0.34	8.10	1.25
59	1	0	0.96	0.04	1	0	0.58	0.42	8.82	10.22
60	1	0	0.91	0.09	1	0	0.6	0.4	8.74	9.21
**Classification accuracy**	**100%**		**100%**		**100%**		**95%**			
**Precision**	**1**		**1**		**1**		**0.95**			
**Recall**	**1**		**1**		**1**		**0.95**			
**F1 score**	**1**		**1**		**1**		**0.95**			
**Mean**									**8.39**	**6.71**
**Std (±)**									**0.69**	**7.29**

Supplementary Table 3. Summary of predicted probability for individual classes and overall classification accuracy of 4 classification models and predicted absolute values (chain length) for the regression model using peak parameters extracted from 60 SERS spectra from 60 $GlcCer_{12}$ blind test samples. Errors in classification are highlighted in red.

**$GlcCer_{12}$**										
ML model	Classification model 1		Classification model 2		Classification model 3		Classification model 4		Regression model 5	
Sample no.	Loaded	Blank	Cerebroside	Monosaccharide	Saturated	Unsaturated	GlcCer	GalCer	Predicted chain length	Difference (%)
1	1	0	0.81	0.19	0.98	0.02	0.52	0.48	12.90	7.48
2	1	0	0.79	0.21	0.98	0.02	0.66	0.34	12.10	0.84
3	1	0	0.85	0.15	0.99	0.01	0.55	0.45	12.84	7.00
4	1	0	0.79	0.21	0.99	0.01	0.69	0.31	11.80	1.67
5	1	0	0.87	0.13	1	0	0.6	0.4	11.90	0.83
6	1	0	0.85	0.15	0.98	0.02	0.4	0.6*	12.66	5.50
7	1	0	0.7	0.3	0.87	0.13	0.64	0.36	11.92	0.65
8	1	0	0.83	0.17	0.98	0.02	0.83	0.17	12.10	0.83
9	1	0	0.75	0.25	0.98	0.02	0.54	0.46	12.10	0.83
10	1	0	0.84	0.16	0.99	0.01	0.51	0.49	13.16	9.64
11	1	0	0.83	0.17	0.99	0.01	0.52	0.48	11.84	1.36
12	1	0	0.77	0.23	0.99	0.01	0.82	0.18	12.10	0.84
13	1	0	0.78	0.22	0.95	0.05	0.55	0.45	12.10	0.83
14	1	0	0.78	0.22	0.97	0.03	0.7	0.3	13.10	9.18
15	1	0	0.86	0.14	0.99	0.01	0.48	0.52*	12.10	0.83
16	1	0	0.76	0.24	0.96	0.04	0.51	0.49	11.90	0.83
17	1	0	0.75	0.25	0.98	0.02	0.61	0.39	12.29	2.38
18	1	0	0.76	0.24	0.99	0.01	0.51	0.49	12.09	0.75
19	1	0	0.81	0.19	0.98	0.02	0.81	0.19	12.10	0.84
20	1	0	0.84	0.16	1	0	0.7	0.3	11.90	0.83
21	1	0	0.79	0.21	0.98	0.02	0.51	0.49	11.81	1.59
22	1	0	0.63	0.37	0.95	0.05	0.63	0.37	10.21	14.94
23	1	0	0.66	0.34	0.95	0.05	0.66	0.34	12.07	0.60
24	1	0	0.69	0.31	0.98	0.02	0.71	0.29	11.33	5.56
25	1	0	0.7	0.3	0.99	0.01	0.79	0.21	11.54	3.81
26	1	0	0.58	0.42	0.93	0.07	0.59	0.41	11.90	0.83
27	1	0	0.84	0.16	0.98	0.02	0.53	0.47	11.07	7.77
28	1	0	0.86	0.14	0.97	0.03	0.54	0.46	11.90	0.83
29	1	0	0.85	0.15	0.99	0.01	0.51	0.49	11.38	5.18
30	1	0	0.89	0.11	0.99	0.01	0.73	0.27	12.10	0.83
31	1	0	0.78	0.22	1	0	0.69	0.31	11.90	0.84
32	1	0	0.72	0.28	0.99	0.01	0.69	0.31	12.17	1.39
33	1	0	0.84	0.16	1	0	0.67	0.33	10.73	10.60
34	1	0	0.8	0.2	0.99	0.01	0.54	0.46	11.71	2.39
35	1	0	0.82	0.18	1	0	0.55	0.45	12.10	0.83
36	1	0	0.77	0.23	0.98	0.02	0.51	0.49	11.36	5.34
37	1	0	0.62	0.38	0.99	0.01	0.8	0.2	11.61	3.25
38	1	0	0.55	0.45	0.92	0.08	0.76	0.24	11.28	6.01
39	1	0	0.65	0.35	0.98	0.02	0.46	0.54*	11.11	7.39
40	1	0	0.84	0.16	0.96	0.04	0.8	0.2	11.97	0.26
41	1	0	0.85	0.15	0.98	0.02	0.53	0.47	11.90	0.84
42	1	0	0.78	0.22	0.88	0.12	0.56	0.44	12.10	0.83
43	1	0	0.81	0.19	0.99	0.01	0.53	0.47	12.10	0.83
44	1	0	0.85	0.15	0.96	0.04	0.44	0.56*	12.08	0.71
45	1	0	0.85	0.15	0.95	0.05	0.64	0.36	11.66	2.86
46	1	0	0.8	0.2	0.92	0.08	0.7	0.3	11.79	1.74

47	1	0	0.77	0.23	0.93	0.07	0.77	0.23	12.06	0.52
48	1	0	0.76	0.24	0.85	0.15	0.55	0.45	12.48	4.00
49	1	0	0.82	0.18	0.95	0.05	0.47	0.53*	12.10	0.83
50	1	0	0.83	0.17	0.93	0.07	0.52	0.48	11.13	7.21
51	1	0	0.84	0.16	0.91	0.09	0.62	0.38	15.84	32.00
52	1	0	0.66	0.34	0.94	0.06	0.77	0.23	11.72	2.36
53	1	0	0.75	0.25	0.92	0.08	0.57	0.43	12.51	4.21
54	1	0	0.73	0.27	0.98	0.02	0.79	0.21	12.35	2.92
55	1	0	0.83	0.17	0.95	0.05	0.61	0.39	11.86	1.20
56	1	0	0.75	0.25	0.9	0.1	0.76	0.24	12.10	0.83
57	1	0	0.8	0.2	0.99	0.01	0.74	0.26	10.50	12.46
58	1	0	0.74	0.26	0.87	0.13	0.64	0.36	11.72	2.36
59	1	0	0.88	0.12	1	0	0.85	0.15	8.69	27.58
60	1	0	0.85	0.15	0.86	0.14	0.51	0.49	12.25	2.07
**Classification accuracy**	**100%**		**100%**		**100%**		**92%**			
**Precision**	**1**		**1**		**1**		**0.92**			
**Recall**	**1**		**1**		**1**		**0.92**			
**F1 score**	**1**		**1**		**1**		**0.92**		**-**	
**Mean**									**11.92**	**4.04**
**Std (±)**									**0.85**	**5.81**

Supplementary Table 4. Summary of predicted probability for individual classes and overall classification accuracy of 4 classification models and predicted absolute values (chain length) for the regression model using peak parameters extracted from 60 SERS spectra from 60 $GlcCer_{16}$ blind test samples. Errors in classification are highlighted in red.

**$GlcCer_{16}$**										
ML model	Classification model 1		Classification model 2		Classification model 3		Classification model 4		Regression model 5	
Sample no.	Loaded	Blank	Cerebroside	Monosaccharide	Saturated	Unsaturated	GlcCer	GalCer	Predicted chain length	Difference (%)
1	1	0	0.89	0.11	1	0	0.85	0.15	15.90	0.62
2	1	0	0.94	0.06	1	0	0.84	0.16	15.90	0.63
3	1	0	0.97	0.03	0.99	0.01	0.84	0.16	16.10	0.63
4	1	0	0.91	0.09	1	0	0.89	0.11	16.04	0.25
5	1	0	0.93	0.07	0.96	0.04	0.78	0.22	15.90	0.63
6	1	0	0.9	0.1	0.99	0.01	0.85	0.15	15.59	2.56
7	1	0	0.93	0.07	1	0	0.86	0.14	15.95	0.34
8	1	0	0.94	0.06	1	0	0.84	0.16	15.90	0.63
9	1	0	0.96	0.04	1	0	0.85	0.15	15.90	0.62
10	1	0	0.93	0.07	0.99	0.01	0.84	0.16	16.07	0.44
11	1	0	0.96	0.04	1	0	0.81	0.19	15.97	0.21
12	1	0	0.93	0.07	0.98	0.02	0.85	0.15	15.94	0.35
13	1	0	0.94	0.06	0.98	0.02	0.87	0.13	16.34	2.13
14	1	0	0.94	0.06	1	0	0.76	0.24	15.90	0.62
15	1	0	0.9	0.1	1	0	0.87	0.13	16.08	0.48
16	1	0	1	0	1	0	0.84	0.16	16.24	1.52
17	1	0	1	0	1	0	0.74	0.26	16.10	0.63
18	1	0	0.98	0.02	1	0	0.85	0.15	15.90	0.62
19	1	0	0.98	0.02	1	0	0.73	0.27	15.86	0.87
20	1	0	0.98	0.02	1	0	0.82	0.18	15.90	0.63
21	1	0	0.99	0.01	1	0	0.73	0.27	16.78	4.88
22	1	0	0.99	0.01	1	0	0.73	0.27	16.72	4.48
23	1	0	1	0	1	0	0.78	0.22	16.87	5.44
24	1	0	0.99	0.01	1	0	0.69	0.31	15.71	1.84
25	1	0	0.99	0.01	1	0	0.77	0.23	16.24	1.53
26	1	0	0.99	0.01	1	0	0.77	0.23	15.90	0.62
27	1	0	0.97	0.03	1	0	0.77	0.23	16.14	0.89
28	1	0	0.99	0.01	1	0	0.71	0.29	15.32	4.25
29	1	0	0.99	0.01	1	0	0.78	0.22	16.15	0.96
30	1	0	0.99	0.01	1	0	0.81	0.19	16.10	0.62
31	1	0	0.99	0.01	1	0	0.8	0.2	14.26	10.91
32	1	0	0.98	0.02	1	0	0.81	0.19	15.49	3.20
33	1	0	0.99	0.01	1	0	0.77	0.23	15.72	1.73
34	1	0	1	0	1	0	0.81	0.19	16.10	0.62
35	1	0	0.9	0.1	0.98	0.02	0.76	0.24	15.90	0.63
36	1	0	0.96	0.04	1	0	0.75	0.25	15.90	0.62
37	1	0	0.98	0.02	1	0	0.72	0.28	17.00	6.26
38	1	0	0.98	0.02	1	0	0.8	0.2	16.10	0.63
39	1	0	0.93	0.07	1	0	0.78	0.22	16.19	1.20
40	1	0	0.98	0.02	1	0	0.78	0.22	16.34	2.10
41	1	0	0.98	0.02	1	0	0.83	0.17	15.90	0.65
42	1	0	0.98	0.02	0.99	0.01	0.65	0.35	15.79	1.30
43	1	0	0.98	0.02	1	0	0.75	0.25	16.10	0.63
44	1	0	0.99	0.01	1	0	0.72	0.28	16.10	0.63
45	1	0	0.99	0.01	0.99	0.01	0.69	0.31	16.10	0.62

46	1	0	0.98	0.02	1	0	0.77	0.23	15.90	0.63
47	1	0	1	0	1	0	0.81	0.19	15.86	0.86
48	1	0	0.98	0.02	1	0	0.8	0.2	15.90	0.63
49	1	0	0.99	0.01	1	0	0.78	0.22	15.63	2.29
50	1	0	0.95	0.05	1	0	0.82	0.18	15.90	0.63
51	1	0	0.97	0.03	1	0	0.8	0.2	16.10	0.63
52	1	0	0.99	0.01	1	0	0.78	0.22	16.04	0.28
53	1	0	0.99	0.01	1	0	0.77	0.23	16.10	0.62
54	1	0	0.99	0.01	1	0	0.8	0.2	15.64	2.25
55	1	0	0.95	0.05	1	0	0.82	0.18	16.30	1.89
56	1	0	0.95	0.05	0.99	0.01	0.8	0.2	16.10	0.63
57	1	0	0.94	0.06	0.99	0.01	0.81	0.19	16.34	2.11
58	1	0	0.93	0.07	1	0	0.8	0.2	16.05	0.28
59	1	0	0.91	0.09	0.97	0.03	0.77	0.23	15.90	0.63
60	1	0	0.94	0.06	0.99	0.01	0.79	0.21	15.90	0.63
**Classification accuracy**	**100%**		**100%**		**100%**		**100%**			
**Precision**	**1**		**1**		**1**		**1**			
**Recall**	**1**		**1**		**1**		**1**			
**F1 score**	**1**		**1**		**1**		**1**		**-**	
**Mean**									**16.00**	**1.46**
**Std (±)**									**0.37**	**1.80**

Supplementary Table 5. Summary of predicted probability for individual classes and overall classification accuracy of 4 classification models and predicted absolute values (chain length) for the regression model using peak parameters extracted from 60 SERS spectra from 60 $GlcCer_{18}$ blind test samples. Errors in classification are highlighted in red.

**$GlcCer_{18}$**										
ML model	Classification model 1		Classification model 2		Classification model 3		Classification model 4		Regression model 5	
Sample no.	Loaded	Blank	Cerebroside	Monosaccharide	Saturated	Unsaturated	GlcCer	GalCer	Predicted chain length	Difference (%)
1	1	0	0.98	0.02	1	0	0.48	0.52*	17.90	0.55
2	1	0	0.99	0.01	1	0	0.59	0.41	18.20	1.10
3	1	0	0.99	0.01	1	0	0.45	0.55*	17.90	0.56
4	1	0	1	0	1	0	0.53	0.47	18.10	0.56
5	1	0	0.99	0.01	1	0	0.61	0.39	17.90	0.56
6	1	0	0.98	0.02	0.99	0.01	0.63	0.37	17.90	0.56
7	1	0	0.95	0.05	1	0	0.56	0.44	17.92	0.46
8	1	0	0.97	0.03	1	0	0.51	0.49	17.90	0.56
9	1	0	0.99	0.01	1	0	0.5	0.5	18.10	0.55
10	1	0	1	0	1	0	0.47	0.53*	18.10	0.56
11	1	0	0.98	0.02	1	0	0.68	0.32	17.90	0.56
12	1	0	0.99	0.01	1	0	0.58	0.42	18.10	0.56
13	1	0	0.99	0.01	1	0	0.61	0.39	18.05	0.29
14	1	0	0.99	0.01	0.99	0.01	0.66	0.34	18.10	0.56
15	1	0	0.99	0.01	1	0	0.46	0.54*	18.10	0.56
16	1	0	0.99	0.01	1	0	0.51	0.49	17.90	0.56
17	1	0	0.99	0.01	1	0	0.51	0.49	18.07	0.37
18	1	0	0.99	0.01	1	0	0.55	0.45	18.10	0.56
19	1	0	0.99	0.01	1	0	0.52	0.48	17.90	0.56
20	1	0	0.98	0.02	0.99	0.01	0.66	0.34	17.90	0.56
21	1	0	0.99	0.01	1	0	0.55	0.45	17.95	0.27
22	1	0	0.93	0.07	1	0	0.67	0.33	18.10	0.56
23	1	0	0.97	0.03	1	0	0.63	0.37	17.90	0.55
24	1	0	0.91	0.09	0.96	0.04	0.52	0.48	18.10	0.56
25	1	0	1	0	0.99	0.01	0.54	0.46	18.10	0.55
26	1	0	0.99	0.01	1	0	0.51	0.49	17.90	0.56
27	1	0	1	0	1	0	0.52	0.48	18.10	0.56
28	1	0	1	0	0.99	0.01	0.55	0.45	18.09	0.48
29	1	0	0.98	0.02	0.99	0.01	0.54	0.46	18.10	0.56
30	1	0	0.98	0.02	0.99	0.01	0.59	0.41	17.90	0.56
31	1	0	0.99	0.01	1	0	0.53	0.47	17.90	0.56
32	1	0	0.92	0.08	0.96	0.04	0.51	0.49	17.90	0.55
33	1	0	0.99	0.01	1	0	0.59	0.41	18.10	0.56
34	1	0	0.99	0.01	1	0	0.51	0.49	18.10	0.56
35	1	0	0.99	0.01	1	0	0.61	0.39	18.10	0.55
36	1	0	0.96	0.04	1	0	0.64	0.36	17.90	0.56
37	1	0	0.93	0.07	1	0	0.53	0.47	15.30	14.99
38	1	0	0.98	0.02	1	0	0.56	0.44	17.90	0.56
39	1	0	0.94	0.06	0.96	0.04	0.45	0.55*	17.90	0.56
40	1	0	0.99	0.01	0.99	0.01	0.6	0.4	17.90	0.56
41	1	0	0.99	0.01	1	0	0.64	0.36	18.10	0.56
42	1	0	0.96	0.04	1	0	0.51	0.49	17.90	0.56
43	1	0	0.99	0.01	0.99	0.01	0.59	0.41	18.10	0.56
44	1	0	0.99	0.01	0.99	0.01	0.58	0.42	17.90	0.56
45	1	0	0.98	0.02	0.99	0.01	0.57	0.43	18.10	0.56
46	1	0	0.98	0.02	1	0	0.49	0.51*	17.92	0.47

47	1	0	0.99	0.01	1	0	0.52	0.48	17.69	1.70
48	1	0	1	0	1	0	0.54	0.46	18.10	0.56
49	1	0	0.99	0.01	0.99	0.01	0.65	0.35	17.90	0.56
50	1	0	1	0	1	0	0.55	0.45	17.90	0.56
51	1	0	0.99	0.01	0.99	0.01	0.58	0.42	18.10	0.56
52	1	0	0.93	0.07	1	0	0.52	0.48	17.90	0.56
53	1	0	0.98	0.02	1	0	0.56	0.44	17.90	0.56
54	1	0	0.96	0.04	1	0	0.54	0.46	18.10	0.56
55	1	0	0.99	0.01	0.98	0.02	0.64	0.36	18.10	0.56
56	1	0	0.98	0.02	0.99	0.01	0.7	0.3	17.90	0.56
57	1	0	0.99	0.01	1	0	0.6	0.4	17.90	0.56
58	1	0	0.97	0.03	0.99	0.01	0.73	0.27	17.90	0.56
59	1	0	0.99	0.01	0.99	0.01	0.62	0.38	17.90	0.56
60	1	0	0.98	0.02	1	0	0.68	0.32	17.90	0.56
**Classification accuracy**	100%		100%		100%		90%			
**Precision**	**1**		**1**		**1**		**0.91**			
**Recall**	**1**		**1**		**1**		**0.91**			
**F1 score**	**1**		**1**		**1**		**0.91**			
**Mean**									17.94	0.81
**Std (±)**									0.36	1.85

Supplementary Table 6. Summary of predicted probability for individual classes and overall classification accuracy of 4 classification models using peak parameters extracted from 60 SERS spectra from 60 $GlcCer_{24:1}$ blind test samples. Errors in classification are highlighted in red.

**$GlcCer_{24:1}$**								
ML model	Classification model 1		Classification model 2		Classification model 3		Classification model 4	
Sample no.	Loaded	Blank	Cerebroside	Monosaccharide	Saturated	Unsaturated	GlcCer	GalCer
1	1	0	0.55	0.45	0.3	0.7	0.71	0.29
2	1	0	0.69	0.31	0.31	0.69	0.63	0.37
3	1	0	0.63	0.37	0.35	0.65	0.63	0.37
4	1	0	0.75	0.25	0.46	0.54	0.7	0.3
5	1	0	0.72	0.28	0.4	0.6	0.66	0.34
6	1	0	0.81	0.19	0.36	0.64	0.64	0.36
7	1	0	0.74	0.26	0.41	0.59	0.61	0.39
8	1	0	0.61	0.39	0.34	0.66	0.77	0.23
9	1	0	0.55	0.45	0.38	0.62	0.66	0.34
10	1	0	0.42	0.58*	0.42	0.58	0.74	0.26
11	1	0	0.61	0.39	0.43	0.57	0.76	0.24
12	1	0	0.67	0.33	0.37	0.63	0.63	0.37
13	1	0	0.61	0.39	0.38	0.62	0.57	0.43
14	1	0	0.54	0.46	0.42	0.58	0.72	0.28
15	1	0	0.67	0.33	0.42	0.58	0.67	0.33
16	1	0	0.79	0.21	0.49	0.51	0.66	0.34
17	1	0	0.78	0.22	0.66	0.34	0.66	0.34
18	1	0	0.77	0.23	0.37	0.63	0.74	0.26
19	1	0	0.79	0.21	0.48	0.52	0.72	0.28
20	1	0	0.73	0.27	0.41	0.59	0.67	0.33
21	1	0	0.46	0.54*	0.37	0.63	0.64	0.36
22	1	0	0.71	0.29	0.33	0.67	0.77	0.23
23	1	0	0.7	0.3	0.56	0.44	0.63	0.37
24	1	0	0.66	0.34	0.49	0.51	0.76	0.24
25	1	0	0.44	0.56*	0.3	0.7	0.66	0.34
26	1	0	0.67	0.33	0.38	0.62	0.66	0.34
27	1	0	0.56	0.44	0.4	0.6	0.73	0.27
28	1	0	0.66	0.34	0.31	0.69	0.78	0.22
29	1	0	0.7	0.3	0.48	0.52	0.64	0.36
30	1	0	0.77	0.23	0.38	0.62	0.75	0.25
31	1	0	0.58	0.42	0.41	0.59	0.71	0.29
32	1	0	0.87	0.13	0.29	0.71	0.71	0.29
33	1	0	0.83	0.17	0.31	0.69	0.77	0.23
34	1	0	0.59	0.41	0.51	0.49	0.66	0.34
35	1	0	0.93	0.07	0.43	0.57	0.73	0.27
36	1	0	0.77	0.23	0.35	0.65	0.66	0.34
37	1	0	0.87	0.13	0.37	0.63	0.71	0.29
38	1	0	0.56	0.44	0.34	0.66	0.6	0.4
39	1	0	0.64	0.36	0.51	0.49	0.8	0.2
40	1	0	0.71	0.29	0.34	0.66	0.73	0.27
41	1	0	0.71	0.29	0.41	0.59	0.69	0.31
42	1	0	0.61	0.39	0.44	0.56	0.73	0.27
43	1	0	0.46	0.54*	0.4	0.6	0.68	0.32
44	1	0	0.79	0.21	0.37	0.63	0.69	0.31
45	1	0	0.62	0.38	0.35	0.65	0.74	0.26
46	1	0	0.7	0.3	0.41	0.59	0.75	0.25
47	1	0	0.81	0.19	0.31	0.69	0.8	0.2
48	1	0	0.71	0.29	0.4	0.6	0.74	0.26

49	1	0	0.53	0.47	0.53	0.47	0.64	0.36
50	1	0	0.6	0.4	0.64	0.36	0.66	0.34
51	1	0	0.6	0.4	0.46	0.54	0.71	0.29
52	1	0	0.78	0.22	0.25	0.75	0.75	0.25
53	1	0	0.65	0.35	0.34	0.66	0.73	0.27
54	1	0	0.75	0.25	0.46	0.54	0.7	0.3
55	1	0	0.77	0.23	0.37	0.63	0.74	0.26
56	1	0	0.62	0.38	0.35	0.65	0.62	0.38
57	1	0	0.67	0.33	0.38	0.62	0.66	0.34
58	1	0	0.75	0.25	0.38	0.62	0.62	0.38
59	1	0	0.66	0.34	0.43	0.57	0.65	0.35
60	1	0	0.67	0.33	0.33	0.67	0.63	0.37
**Classification accuracy**	**100%**		**93%**		**90%**		**100%**	
**Precision**	**1**		**0.94**		**0.91**		**1**	
**Recall**	**1**		**0.94**		**0.91**		**1**	
**F1 score**	**1**		**0.94**		**0.91**		**1**	

Supplementary Table 7. Summary of predicted probability for individual classes and overall classification accuracy of 4 classification models and predicted absolute values (chain length) for the regression model using peak parameters extracted from 60 SERS spectra from 60 $GalCer_8$ blind test samples. Errors in classification are highlighted in red. Errors in classification are highlighted in red.

**$GalCer_8$**										
ML model	Classification model 1		Classification model 2		Classification model 3		Classification model 4		Regression model 5	
Sample no.	Loaded	Blank	Cerebroside	Monosaccharide	Saturated	Unsaturated	0.36	0.64	Predicted chain length	Difference (%)
1	1	0	0.75	0.25	0.93	0.07	0.44	0.56	9.25	15.68
2	1	0	0.89	0.11	0.96	0.04	0.34	0.66	8.80	10.03
3	1	0	0.81	0.19	0.96	0.04	0.43	0.57	8.19	2.43
4	1	0	0.97	0.03	0.98	0.02	0.34	0.66	7.67	4.15
5	1	0	0.95	0.05	0.94	0.06	0.39	0.61	8.00	0.04
6	1	0	0.95	0.05	0.98	0.02	0.43	0.57	9.72	21.49
7	1	0	0.61	0.39	0.88	0.12	0.39	0.61	8.68	8.45
8	1	0	0.88	0.12	0.99	0.01	0.43	0.57	8.15	1.90
9	1	0	0.91	0.09	0.99	0.01	0.36	0.64	7.66	4.28
10	1	0	0.94	0.06	0.97	0.03	0.44	0.56	9.33	16.57
11	1	0	0.96	0.04	0.99	0.01	0.46	0.54	8.75	9.39
12	1	0	0.91	0.09	0.96	0.04	0.33	0.67	9.61	20.16
13	1	0	0.9	0.1	0.97	0.03	0.42	0.58	8.40	4.94
14	1	0	0.93	0.07	0.93	0.07	0.39	0.61	8.65	8.08
15	1	0	0.88	0.12	0.97	0.03	0.43	0.57	10.25	28.12
16	1	0	0.91	0.09	0.98	0.02	0.33	0.67	9.99	24.88
17	1	0	0.89	0.11	0.98	0.02	0.34	0.66	8.75	9.34
18	1	0	0.91	0.09	0.97	0.03	0.34	0.66	8.69	8.67
19	1	0	0.96	0.04	0.99	0.01	0.31	0.69	8.12	1.56
20	1	0	0.96	0.04	0.99	0.01	0.3	0.7	8.70	8.74
21	1	0	0.9	0.1	0.98	0.02	0.34	0.66	9.17	14.59
22	1	0	0.83	0.17	0.97	0.03	0.41	0.59	9.71	21.35
23	1	0	0.89	0.11	0.91	0.09	0.41	0.59	9.27	15.84
24	1	0	0.89	0.11	0.99	0.01	0.36	0.64	8.61	7.65
25	1	0	0.95	0.05	0.97	0.03	0.39	0.61	8.33	4.15
26	1	0	0.92	0.08	0.99	0.01	0.28	0.72	8.13	1.66
27	1	0	0.94	0.06	0.98	0.02	0.36	0.64	7.72	3.51
28	1	0	0.94	0.06	0.93	0.07	0.3	0.7	8.79	9.82
29	1	0	0.95	0.05	0.98	0.02	0.33	0.67	10.54	31.76
30	1	0	0.94	0.06	0.99	0.01	0.25	0.75	10.62	32.72
31	1	0	0.95	0.05	0.99	0.01	0.25	0.75	9.61	20.12
32	1	0	0.95	0.05	1	0	0.2	0.8	10.84	35.50
33	1	0	0.94	0.06	0.99	0.01	0.27	0.73	8.14	1.76
34	1	0	0.97	0.03	0.99	0.01	0.33	0.67	8.74	9.28
35	1	0	0.85	0.15	0.91	0.09	0.3	0.7	8.74	9.28
36	1	0	0.91	0.09	0.94	0.06	0.43	0.57	9.20	15.01
37	1	0	0.86	0.14	0.99	0.01	0.28	0.72	9.94	24.31
38	1	0	0.91	0.09	1	0	0.44	0.56	9.44	18.04

39	1	0	0.93	0.07	0.99	0.01	0.37	0.63	10.86	35.73
40	1	0	0.98	0.02	0.99	0.01	0.3	0.7	10.20	27.55
41	1	0	0.98	0.02	0.99	0.01	0.32	0.68	8.05	0.57
42	1	0	0.96	0.04	0.98	0.02	0.24	0.76	8.52	6.48
43	1	0	0.98	0.02	0.99	0.01	0.22	0.78	8.15	1.81
44	1	0	0.83	0.17	0.97	0.03	0.25	0.75	8.21	2.63
45	1	0	0.96	0.04	0.99	0.01	0.41	0.59	8.24	3.05
46	1	0	0.95	0.05	0.99	0.01	0.37	0.63	7.11	11.14
47	1	0	0.94	0.06	0.99	0.01	0.31	0.69	7.33	8.40
48	1	0	0.97	0.03	0.99	0.01	0.39	0.61	8.62	7.76
49	1	0	0.98	0.02	0.99	0.01	0.38	0.62	8.74	9.28
50	1	0	0.86	0.14	0.95	0.05	0.36	0.64	9.68	20.99
51	1	0	0.8	0.2	0.97	0.03	0.41	0.59	10.51	31.42
52	1	0	0.76	0.24	0.92	0.08	0.28	0.72	8.37	4.66
53	1	0	0.89	0.11	0.99	0.01	0.35	0.65	8.58	7.24
54	1	0	0.87	0.13	0.98	0.02	0.36	0.64	8.12	1.44
55	1	0	0.96	0.04	0.99	0.01	0.41	0.59	7.66	4.24
56	1	0	0.95	0.05	0.98	0.02	0.34	0.66	8.48	5.94
57	1	0	0.93	0.07	0.97	0.03	0.36	0.64	8.26	3.29
58	1	0	0.97	0.03	0.98	0.02	0.34	0.66	7.82	2.19
59	1	0	0.95	0.05	0.98	0.02	0.34	0.66	8.31	3.84
60	1	0	0.94	0.06	0.98	0.02	0.36	0.64	8.68	8.45
**Classification accuracy**	**100%**		**100%**		**100%**		**100%**		-	
**Precision**	**1**		**1**		**1**		**1**			
**Recall**	**1**		**1**		**1**		**1**			
**F1 score**	**1**		**1**		**1**		**1**			
**Mean**									**8.82**	**11.56**
**Std (±)**									**0.89**	**9.80**

Supplementary Table 8. Summary of predicted probability for individual classes and overall classification accuracy of 4 classification models and predicted absolute values (chain length) for the regression model using peak parameters extracted from 60 SERS spectra from 60 $GalCer_{12}$ blind test samples. Errors in classification are highlighted in red.

**$GalCer_{12}$**										
ML model	Classification model 1		Classification model 2		Classification model 3		Classification model 4		Regression model 5	
Sample no.	Loaded	Blank	Cerebroside	Monosaccharide	Saturated	Unsaturated	GlcCer	GalCer	Predicted chain length	Difference (%)
1	1	0	0.98	0.02	0.96	0.04	0.46	0.54	11.60	3.32
2	1	0	0.97	0.03	0.94	0.06	0.39	0.61	13.15	9.55
3	1	0	0.94	0.06	0.88	0.12	0.41	0.59	1.79	85.09
4	1	0	0.94	0.06	0.95	0.05	0.43	0.57	12.75	6.27
5	1	0	0.97	0.03	0.96	0.04	0.43	0.57	13.97	16.41
6	1	0	0.98	0.02	0.93	0.07	0.38	0.62	14.80	23.32
7	1	0	0.95	0.05	0.91	0.09	0.4	0.6	12.17	1.38
8	1	0	0.99	0.01	0.97	0.03	0.44	0.56	12.63	5.22
9	1	0	0.98	0.02	0.96	0.04	0.39	0.61	14.43	20.23
10	1	0	0.98	0.02	0.95	0.05	0.42	0.58	13.16	9.65
11	1	0	0.91	0.09	0.89	0.11	0.38	0.62	7.90	34.16
12	1	0	0.98	0.02	0.96	0.04	0.44	0.56	14.54	21.13
13	1	0	0.98	0.02	0.94	0.06	0.39	0.61	10.04	16.33
14	1	0	0.98	0.02	0.94	0.06	0.41	0.59	13.77	14.79
15	1	0	0.97	0.03	0.91	0.09	0.28	0.72	11.92	0.71
16	1	0	0.96	0.04	0.98	0.02	0.47	0.53	11.29	5.92
17	1	0	0.95	0.05	0.96	0.04	0.46	0.54	14.29	19.09
18	1	0	0.96	0.04	0.92	0.08	0.44	0.56	13.81	15.08
19	1	0	0.96	0.04	0.92	0.08	0.39	0.61	15.31	27.60
20	1	0	0.96	0.04	0.88	0.12	0.36	0.64	14.54	21.21
21	1	0	0.97	0.03	0.89	0.11	0.43	0.57	14.56	21.30
22	1	0	0.97	0.03	0.98	0.02	0.36	0.64	11.39	5.05
23	1	0	0.94	0.06	0.98	0.02	0.41	0.59	12.50	4.19
24	1	0	0.96	0.04	0.98	0.02	0.48	0.52	14.86	23.80
25	1	0	0.96	0.04	0.91	0.09	0.39	0.61	12.98	8.13
26	1	0	0.95	0.05	0.87	0.13	0.35	0.65	13.81	15.11
27	1	0	0.95	0.05	0.93	0.07	0.43	0.57	15.25	27.06
28	1	0	0.95	0.05	0.88	0.12	0.4	0.6	13.25	10.44
29	1	0	0.96	0.04	0.87	0.13	0.33	0.67	12.46	3.84
30	1	0	0.95	0.05	0.86	0.14	0.35	0.65	12.38	3.19
31	1	0	0.98	0.02	0.98	0.02	0.42	0.58	14.12	17.70
32	1	0	0.95	0.05	0.89	0.11	0.38	0.62	12.23	1.94
33	1	0	0.99	0.01	0.97	0.03	0.42	0.58	14.50	20.80
34	1	0	0.98	0.02	0.96	0.04	0.38	0.62	11.46	4.47
35	1	0	0.96	0.04	0.94	0.06	0.41	0.59	11.91	0.74
36	1	0	0.96	0.04	0.96	0.04	0.39	0.61	11.98	0.18
37	1	0	0.98	0.02	0.99	0.01	0.36	0.64	11.33	5.57
38	1	0	0.98	0.02	0.96	0.04	0.38	0.62	11.24	6.32
39	1	0	0.82	0.18	0.93	0.07	0.47	0.53	11.33	5.56
40	1	0	0.97	0.03	0.95	0.05	0.48	0.52	11.53	3.88
41	1	0	0.98	0.02	0.93	0.07	0.44	0.56	12.55	4.58
42	1	0	0.96	0.04	0.95	0.05	0.46	0.54	15.51	29.25
43	1	0	0.98	0.02	0.92	0.08	0.41	0.59	12.23	1.95
44	1	0	0.99	0.01	0.94	0.06	0.4	0.6	12.85	7.12

45	1	0	0.98	0.02	0.89	0.11	0.4	0.6	13.18	9.80
46	1	0	0.97	0.03	0.96	0.04	0.47	0.53	11.37	5.26
47	1	0	0.94	0.06	0.95	0.05	0.36	0.64	13.30	10.87
48	1	0	0.97	0.03	0.94	0.06	0.35	0.65	13.05	8.77
49	1	0	0.95	0.05	0.85	0.15	0.4	0.6	15.21	26.72
50	1	0	0.98	0.02	0.93	0.07	0.35	0.65	11.79	1.75
51	1	0	0.94	0.06	0.91	0.09	0.34	0.66	13.13	9.39
52	1	0	0.98	0.02	0.97	0.03	0.31	0.69	12.08	0.64
53	1	0	0.96	0.04	0.96	0.04	0.46	0.54	11.59	3.41
54	1	0	0.96	0.04	0.96	0.04	0.43	0.57	11.86	1.20
55	1	0	0.96	0.04	0.93	0.07	0.42	0.58	12.12	0.98
56	1	0	0.97	0.03	0.93	0.07	0.42	0.58	11.97	0.24
57	1	0	0.97	0.03	0.96	0.04	0.45	0.55	13.63	13.58
58	1	0	0.96	0.04	0.89	0.11	0.4	0.6	9.03	24.75
59	1	0	0.98	0.02	0.92	0.08	0.34	0.66	11.58	3.53
60	1	0	0.98	0.02	0.92	0.08	0.33	0.67	13.21	10.10
**Classification accuracy**	**100%**		**100%**		**100%**		**100%**			
**Precision**	**1**		**1**		**1**		**1**			
**Recall**	**1**		**1**		**1**		**1**			
**F1 score**	**1**		**1**		**1**		**1**		**-**	
**Mean**									**12.57**	**11.99**
**Std (±)**									**2.03**	**12.88**

Supplementary Table 9. Summary of predicted probability for individual classes and overall classification accuracy of 4 classification models and predicted absolute values (chain length) for the regression model using peak parameters extracted from 60 SERS spectra from 60 $GalCer_{16}$ blind test samples. Errors in classification are highlighted in red.

**$GalCer_{16}$**										
	Classification model 1		Classification model 2		Classification model 3		Classification model 4		Regression model 5	
Sample no.	Loaded	Blank	Cerebroside	Monosaccharide	Saturated	Unsaturated	GlcCer	GalCer	Predicted chain length	Difference (%)
1	1	0	0.95	0.05	0.99	0.01	0.14	0.86	13.95	12.79
2	1	0	0.9	0.1	0.99	0.01	0.2	0.8	16.26	1.61
3	1	0	0.96	0.04	0.99	0.01	0.18	0.82	16.30	1.87
4	1	0	0.94	0.06	0.99	0.01	0.3	0.7	15.75	1.58
5	1	0	0.98	0.02	1	0	0.19	0.81	17.36	8.50
6	1	0	0.95	0.05	1	0	0.19	0.81	17.17	7.29
7	1	0	0.83	0.17	1	0	0.14	0.86	17.71	10.71
8	1	0	0.95	0.05	0.99	0.01	0.18	0.82	16.91	5.70
9	1	0	0.9	0.1	1	0	0.17	0.83	15.96	0.25
10	1	0	0.99	0.01	1	0	0.14	0.86	16.08	0.48
11	1	0	0.98	0.02	1	0	0.14	0.86	16.93	5.84
12	1	0	0.99	0.01	1	0	0.3	0.7	16.91	5.69
13	1	0	0.95	0.05	1	0	0.15	0.85	15.90	0.65
14	1	0	0.99	0.01	1	0	0.32	0.68	16.56	3.49
15	1	0	0.96	0.04	1	0	0.13	0.87	12.98	18.86
16	1	0	0.97	0.03	1	0	0.1	0.9	15.90	0.60
17	1	0	0.96	0.04	1	0	0.15	0.85	16.86	5.40
18	1	0	0.97	0.03	1	0	0.09	0.91	17.49	9.29
19	1	0	0.97	0.03	1	0	0.15	0.85	18.13	13.34
20	1	0	0.96	0.04	1	0	0.06	0.94	15.96	0.22
21	1	0	0.98	0.02	1	0	0.12	0.88	17.23	7.66
22	1	0	0.87	0.13	1	0	0.11	0.89	17.74	10.90
23	1	0	0.92	0.08	1	0	0.17	0.83	16.15	0.95
24	1	0	0.92	0.08	0.98	0.02	0.18	0.82	16.44	2.75
25	1	0	0.99	0.01	1	0	0.07	0.93	16.33	2.05
26	1	0	0.99	0.01	1	0	0.07	0.93	18.02	12.63
27	1	0	0.98	0.02	1	0	0.13	0.87	15.77	1.43
28	1	0	0.98	0.02	1	0	0.15	0.85	17.39	8.72
29	1	0	0.98	0.02	1	0	0.07	0.93	16.66	4.14
30	1	0	0.99	0.01	1	0	0.06	0.94	17.63	10.16
31	1	0	0.98	0.02	1	0	0.11	0.89	15.22	4.86
32	1	0	0.98	0.02	1	0	0.16	0.84	16.53	3.34
33	1	0	0.98	0.02	1	0	0.25	0.75	15.35	4.08
34	1	0	0.96	0.04	0.99	0.01	0.25	0.75	17.14	7.15
35	1	0	0.96	0.04	0.99	0.01	0.1	0.9	15.78	1.38
36	1	0	0.99	0.01	1	0	0.12	0.88	15.86	0.89
37	1	0	0.87	0.13	1	0	0.17	0.83	16.46	2.85
38	1	0	0.97	0.03	1	0	0.07	0.93	15.61	2.44
39	1	0	0.91	0.09	0.99	0.01	0.18	0.82	14.99	6.30
40	1	0	0.97	0.03	1	0	0.08	0.92	16.71	4.43
41	1	0	0.98	0.02	1	0	0.18	0.82	16.20	1.25
42	1	0	1	0	1	0	0.09	0.91	17.31	8.20
43	1	0	0.99	0.01	1	0	0.14	0.86	17.90	11.86
44	1	0	0.97	0.03	1	0	0.25	0.75	17.81	11.32
45	1	0	0.98	0.02	1	0	0.08	0.92	16.90	5.60
46	1	0	0.99	0.01	1	0	0.1	0.9	15.37	3.93

47	1	0	0.98	0.02	1	0	0.07	0.93	16.06	0.35
48	1	0	0.99	0.01	1	0	0.09	0.91	16.69	4.32
49	1	0	0.98	0.02	1	0	0.12	0.88	16.33	2.04
50	1	0	0.97	0.03	1	0	0.12	0.88	16.63	3.95
51	1	0	0.97	0.03	1	0	0.11	0.89	17.17	7.32
52	1	0	0.94	0.06	1	0	0.16	0.84	17.82	11.35
53	1	0	0.98	0.02	1	0	0.12	0.88	16.76	4.76
54	1	0	0.98	0.02	1	0	0.08	0.92	17.63	10.16
55	1	0	0.99	0.01	1	0	0.08	0.92	15.84	1.01
56	1	0	0.98	0.02	1	0	0.11	0.89	17.01	6.33
57	1	0	0.99	0.01	1	0	0.13	0.87	16.96	5.98
58	1	0	0.99	0.01	1	0	0.15	0.85	16.49	3.08
59	1	0	0.98	0.02	1	0	0.1	0.9	15.79	1.33
60	1	0	0.99	0.01	1	0	0.09	0.91	17.63	10.21
**Classification accuracy**	**100%**		**100%**		**100%**		**100%**		-	
**Precision**	**1**		**1**		**1**		**1**			
**Recall**	**1**		**1**		**1**		**1**			
**F1 score**	**1**		**1**		**1**		**1**			
**Mean**									16.54	5.46
**Std (±)**									**0.96**	**4.15**

Supplementary Table 10. Summary of predicted probability for individual classes and overall classification accuracy of 4 classification models and predicted absolute values (chain length) for the regression model using peak parameters extracted from 60 SERS spectra from 60 $GalCer_{18}$ blind test samples. Errors in classification are highlighted in red.

**$GalCer_{18}$**										
ML model	Classification model 1		Classification model 2		Classification model 3		Classification model 4		Regression model 5	
Sample no.	Loaded	Blank	Cerebroside	Monosaccharide	Saturated	Unsaturated	GlcCer	GalCer	Predicted chain length	Difference (%)
1	1	0	0.88	0.12	1	0	0.13	0.87	17.23	4.29
2	1	0	0.72	0.28	1	0	0.21	0.79	18.36	2.02
3	1	0	0.9	0.1	1	0	0.17	0.83	18.01	0.06
4	1	0	0.79	0.21	1	0	0.29	0.71	17.99	0.06
5	1	0	0.93	0.07	1	0	0.16	0.84	18.08	0.45
6	1	0	0.83	0.17	1	0	0.17	0.83	19.85	10.28
7	1	0	0.64	0.36	1	0	0.11	0.89	18.01	0.06
8	1	0	0.89	0.11	1	0	0.18	0.82	17.99	0.06
9	1	0	0.73	0.27	0.99	0.01	0.17	0.83	18.01	0.06
10	1	0	0.92	0.08	1	0	0.13	0.87	18.25	1.38
11	1	0	0.89	0.11	0.99	0.01	0.14	0.86	17.99	0.06
12	1	0	0.93	0.07	0.99	0.01	0.3	0.7	17.99	0.05
13	1	0	0.8	0.2	1	0	0.15	0.85	18.01	0.05
14	1	0	0.94	0.06	1	0	0.33	0.67	17.99	0.06
15	1	0	0.84	0.16	1	0	0.13	0.87	17.99	0.05
16	1	0	0.81	0.19	1	0	0.08	0.92	18.88	4.87
17	1	0	0.81	0.19	1	0	0.14	0.86	18.01	0.05
18	1	0	0.81	0.19	1	0	0.09	0.91	17.75	1.41
19	1	0	0.84	0.16	0.99	0.01	0.15	0.85	17.99	0.06
20	1	0	0.75	0.25	1	0	0.06	0.94	17.99	0.06
21	1	0	0.85	0.15	1	0	0.12	0.88	18.01	0.06
22	1	0	0.66	0.34	1	0	0.1	0.9	18.01	0.06
23	1	0	0.74	0.26	1	0	0.16	0.84	18.01	0.06
24	1	0	0.72	0.28	0.99	0.01	0.16	0.84	19.59	8.82
25	1	0	0.88	0.12	1	0	0.07	0.93	18.01	0.05
26	1	0	0.9	0.1	1	0	0.07	0.93	17.90	0.57
27	1	0	0.89	0.11	1	0	0.14	0.86	18.01	0.06
28	1	0	0.91	0.09	1	0	0.16	0.84	17.67	1.84
29	1	0	0.87	0.13	1	0	0.07	0.93	18.01	0.05
30	1	0	0.91	0.09	1	0	0.07	0.93	17.99	0.06
31	1	0	0.96	0.04	1	0	0.11	0.89	18.01	0.06
32	1	0	0.95	0.05	1	0	0.15	0.85	17.99	0.05
33	1	0	0.92	0.08	1	0	0.27	0.73	17.99	0.06
34	1	0	0.87	0.13	0.95	0.05	0.27	0.73	17.99	0.06
35	1	0	0.86	0.14	0.99	0.01	0.13	0.87	17.99	0.06
36	1	0	0.94	0.06	1	0	0.14	0.86	17.99	0.06
37	1	0	0.68	0.32	1	0	0.21	0.79	17.89	0.59
38	1	0	0.88	0.12	1	0	0.1	0.9	18.01	0.05
39	1	0	0.84	0.16	0.98	0.02	0.19	0.81	17.99	0.06
40	1	0	0.9	0.1	1	0	0.11	0.89	17.11	4.92
41	1	0	0.9	0.1	1	0	0.21	0.79	17.99	0.05
42	1	0	0.98	0.02	1	0	0.1	0.9	18.01	0.06
43	1	0	0.96	0.04	1	0	0.13	0.87	18.16	0.91
44	1	0	0.94	0.06	0.99	0.01	0.26	0.74	17.99	0.06
45	1	0	0.94	0.06	1	0	0.09	0.91	18.01	0.05
46	1	0	0.91	0.09	1	0	0.08	0.92	17.99	0.05

47	1	0	0.93	0.07	1	0	0.08	0.92	18.01	0.06
48	1	0	0.92	0.08	1	0	0.09	0.91	18.01	0.05
49	1	0	0.86	0.14	1	0	0.13	0.87	17.99	0.05
50	1	0	0.86	0.14	1	0	0.12	0.88	18.01	0.05
51	1	0	0.87	0.13	1	0	0.12	0.88	18.01	0.06
52	1	0	0.82	0.18	1	0	0.16	0.84	18.01	0.06
53	1	0	0.89	0.11	1	0	0.13	0.87	17.99	0.06
54	1	0	0.91	0.09	1	0	0.08	0.92	18.07	0.39
55	1	0	0.89	0.11	1	0	0.1	0.9	18.01	0.06
56	1	0	0.92	0.08	1	0	0.13	0.87	18.01	0.06
57	1	0	0.93	0.07	1	0	0.17	0.83	17.69	1.72
58	1	0	0.94	0.06	1	0	0.15	0.85	17.99	0.06
59	1	0	0.9	0.1	1	0	0.1	0.9	17.31	3.83
60	1	0	0.91	0.09	1	0	0.1	0.9	17.99	0.06
**Classification accuracy**	**100%**		**100%**		**100%**		**100%**			
**Precision**	**1**		**1**		**1**		**1**			
**Recall**	**1**		**1**		**1**		**1**			
**F1 score**	**1**		**1**		**1**		**1**		**-**	
**Mean**									**18.03**	**0.85**
**Std (±)**									**0.39**	**1.99**

Supplementary Table 11. Summary of predicted probability for individual classes and overall classification accuracy of 4 classification models and predicted absolute values (chain length) for the regression model using peak parameters extracted from 60 SERS spectra from 60 $GalCer_{24}$ blind test samples. Errors in classification are highlighted in red.

**$GalCer_{24}$**										
ML model	Classification model 1		Classification model 2		Classification model 3		Classification model 4		Regression model 5	
Sample no.	Loaded	Blank	Cerebroside	Monosaccharide	Saturated	Unsaturated	GlcCer	GalCer	Predicted chain length	Difference (%)
1	1	0	0.92	0.08	1	0	0.16	0.84	22.16	7.69
2	1	0	0.95	0.05	1	0	0.32	0.68	24.51	2.13
3	1	0	0.97	0.03	0.97	0.03	0.4	0.6	24.49	2.04
4	1	0	0.95	0.05	0.92	0.08	0.33	0.67	24.51	2.13
5	1	0	0.98	0.02	1	0	0.26	0.74	24.49	2.04
6	1	0	0.94	0.06	1	0	0.24	0.76	24.49	2.04
7	1	0	0.81	0.19	0.89	0.11	0.29	0.71	24.49	2.04
8	1	0	0.94	0.06	1	0	0.34	0.66	24.49	2.04
9	1	0	0.82	0.18	0.99	0.01	0.24	0.76	23.89	0.45
10	1	0	0.98	0.02	0.98	0.02	0.33	0.67	24.49	2.04
11	1	0	0.96	0.04	0.92	0.08	0.41	0.59	24.49	2.04
12	1	0	0.98	0.02	0.97	0.03	0.27	0.73	24.49	2.04
13	1	0	0.96	0.04	0.96	0.04	0.31	0.69	24.49	2.04
14	1	0	0.97	0.03	0.98	0.02	0.39	0.61	24.49	2.04
15	1	0	0.97	0.03	0.98	0.02	0.31	0.69	24.51	2.13
16	1	0	0.91	0.09	0.99	0.01	0.43	0.57	24.49	2.04
17	1	0	0.93	0.07	1	0	0.49	0.51	24.49	2.04
18	1	0	0.96	0.04	1	0	0.37	0.63	24.49	2.04
19	1	0	0.96	0.04	0.95	0.05	0.25	0.75	22.89	4.61
20	1	0	0.97	0.03	1	0	0.47	0.53	24.49	2.04
21	1	0	0.98	0.02	1	0	0.3	0.7	24.51	2.12
22	1	0	0.83	0.17	1	0	0.42	0.58	24.51	2.13
23	1	0	0.85	0.15	1	0	0.29	0.71	24.49	2.04
24	1	0	0.94	0.06	1	0	0.46	0.54	24.51	2.13
25	1	0	0.98	0.02	1	0	0.31	0.69	24.51	2.13
26	1	0	0.98	0.02	1	0	0.34	0.66	24.49	2.04
27	1	0	0.93	0.07	0.96	0.04	0.4	0.6	17.26	28.07
28	1	0	0.97	0.03	0.99	0.01	0.38	0.62	24.49	2.04
29	1	0	0.98	0.02	1	0	0.35	0.65	25.02	4.26
30	1	0	0.98	0.02	0.99	0.01	0.34	0.66	24.49	2.04
31	1	0	0.92	0.08	1	0	0.2	0.8	24.49	2.04
32	1	0	0.88	0.12	1	0	0.24	0.76	24.49	2.04
33	1	0	0.96	0.04	1	0	0.27	0.73	24.49	2.04
34	1	0	0.97	0.03	1	0	0.32	0.68	24.51	2.11
35	1	0	0.97	0.03	1	0	0.32	0.68	24.51	2.13
36	1	0	0.93	0.07	1	0	0.25	0.75	24.51	2.13
37	1	0	0.75	0.25	0.99	0.01	0.31	0.69	24.49	2.04
38	1	0	0.9	0.1	1	0	0.33	0.67	24.49	2.04
39	1	0	0.88	0.12	1	0	0.21	0.79	24.49	2.04
40	1	0	0.96	0.04	1	0	0.24	0.76	25.79	7.47
41	1	0	0.97	0.03	1	0	0.35	0.65	24.51	2.13
42	1	0	0.96	0.04	1	0	0.28	0.72	24.68	2.82
43	1	0	0.96	0.04	1	0	0.38	0.62	24.49	2.04
44	1	0	0.94	0.06	0.95	0.05	0.33	0.67	24.49	2.04
45	1	0	0.92	0.08	0.99	0.01	0.25	0.75	24.49	2.04
46	1	0	0.94	0.06	1	0	0.32	0.68	24.05	0.20

47	1	0	0.91	0.09	1	0	0.2	0.8	24.51	2.13
48	1	0	0.96	0.04	0.98	0.02	0.31	0.69	24.49	2.04
49	1	0	0.96	0.04	1	0	0.32	0.68	24.51	2.13
50	1	0	0.97	0.03	1	0	0.32	0.68	24.49	2.04
51	1	0	0.97	0.03	1	0	0.36	0.64	24.49	2.04
52	1	0	0.68	0.32	0.98	0.02	0.42	0.58	22.86	4.75
53	1	0	0.88	0.12	1	0	0.29	0.71	24.51	2.13
54	1	0	0.9	0.1	1	0	0.35	0.65	24.49	2.04
55	1	0	0.98	0.02	1	0	0.32	0.68	24.49	2.04
56	1	0	0.97	0.03	1	0	0.34	0.66	24.49	2.04
57	1	0	0.93	0.07	0.98	0.02	0.23	0.77	23.83	0.72
58	1	0	0.95	0.05	1	0	0.32	0.68	24.51	2.13
59	1	0	0.94	0.06	0.99	0.01	0.25	0.75	24.49	2.04
60	1	0	0.96	0.04	0.98	0.02	0.33	0.67	24.49	2.04
**Classification accuracy**	**100%**		**100%**		**100%**		**100%**			
**Precision**	**1**		**1**		**1**		**1**			
**Recall**	**1**		**1**		**1**		**1**			
**F1 score**	**1**		**1**		**1**		**1**			**-**
**Mean**									**24.29**	**2.74**
**Std (+)**									**1.03**	**3.50**

Supplementary Table 12. Summary of predicted probability for individual classes and overall classification accuracy of 4 classification models and predicted absolute values (chain length) for the regression model using peak parameters extracted from 60 SERS spectra from 60 GalCer$_{24:1}$ blind test samples. Errors in classification are highlighted in red.

**GalCer$_{24:1}$**								
ML model	Classification model 1		Classification model 2		Classification model 3		Classification model 4	
Sample no.	Loaded	Blank	Cerebroside	Monosaccharide	Saturated	Unsaturated	GlcCer	GalCer
1	1	0	0.73	0.27	0.39	0.61	0.33	0.67
2	1	0	0.78	0.22	0.22	0.78	0.28	0.72
3	1	0	0.8	0.2	0.2	0.8	0.29	0.71
4	1	0	0.79	0.21	0.22	0.78	0.26	0.74
5	1	0	0.78	0.22	0.22	0.78	0.26	0.74
6	1	0	0.79	0.21	0.28	0.72	0.33	0.67
7	1	0	0.83	0.17	0.2	0.8	0.29	0.71
8	1	0	0.73	0.27	0.24	0.76	0.29	0.71
9	1	0	0.84	0.16	0.15	0.85	0.24	0.76
10	1	0	0.84	0.16	0.17	0.83	0.26	0.74
11	1	0	0.81	0.19	0.18	0.82	0.27	0.73
12	1	0	0.79	0.21	0.28	0.72	0.33	0.67
13	1	0	0.81	0.19	0.16	0.84	0.26	0.74
14	1	0	0.84	0.16	0.19	0.81	0.3	0.7
15	1	0	0.81	0.19	0.21	0.79	0.28	0.72
16	1	0	0.81	0.19	0.18	0.82	0.29	0.71
17	1	0	0.72	0.28	0.17	0.83	0.28	0.72
18	1	0	0.81	0.19	0.2	0.8	0.26	0.74
19	1	0	0.83	0.17	0.16	0.84	0.28	0.72
20	1	0	0.81	0.19	0.3	0.7	0.29	0.71
21	1	0	0.78	0.22	0.15	0.85	0.19	0.81
22	1	0	0.77	0.23	0.24	0.76	0.29	0.71
23	1	0	0.8	0.2	0.19	0.81	0.29	0.71
24	1	0	0.74	0.26	0.21	0.79	0.27	0.73
25	1	0	0.72	0.28	0.24	0.76	0.3	0.7
26	1	0	0.7	0.3	0.22	0.78	0.24	0.76
27	1	0	0.81	0.19	0.23	0.77	0.32	0.68
28	1	0	0.74	0.26	0.24	0.76	0.29	0.71
29	1	0	0.8	0.2	0.19	0.81	0.29	0.71
30	1	0	0.74	0.26	0.21	0.79	0.23	0.77
31	1	0	0.77	0.23	0.26	0.74	0.3	0.7
32	1	0	0.73	0.27	0.28	0.72	0.3	0.7
33	1	0	0.72	0.28	0.29	0.71	0.32	0.68
34	1	0	0.77	0.23	0.21	0.79	0.29	0.71
35	1	0	0.72	0.28	0.27	0.73	0.27	0.73
36	1	0	0.7	0.3	0.24	0.76	0.27	0.73
37	1	0	0.74	0.26	0.24	0.76	0.27	0.73
38	1	0	0.7	0.3	0.25	0.75	0.29	0.71
39	1	0	0.69	0.31	0.2	0.8	0.21	0.79
40	1	0	0.81	0.19	0.21	0.79	0.26	0.74
41	1	0	0.83	0.17	0.18	0.82	0.24	0.76
42	1	0	0.73	0.27	0.28	0.72	0.29	0.71
43	1	0	0.8	0.2	0.17	0.83	0.19	0.81
44	1	0	0.67	0.33	0.28	0.72	0.27	0.73
45	1	0	0.83	0.17	0.18	0.82	0.29	0.71
46	1	0	0.72	0.28	0.27	0.73	0.29	0.71
47	1	0	0.72	0.28	0.28	0.72	0.32	0.68
48	1	0	0.71	0.29	0.21	0.79	0.2	0.8

49	1	0	0.74	0.26	0.21	0.79	0.24	0.76
50	1	0	0.76	0.24	0.27	0.73	0.33	0.67
51	1	0	0.72	0.28	0.22	0.78	0.28	0.72
52	1	0	0.7	0.3	0.15	0.85	0.2	0.8
53	1	0	0.74	0.26	0.17	0.83	0.2	0.8
54	1	0	0.73	0.27	0.23	0.77	0.28	0.72
55	1	0	0.74	0.26	0.19	0.81	0.26	0.74
56	1	0	0.79	0.21	0.27	0.73	0.31	0.69
57	1	0	0.7	0.3	0.15	0.85	0.19	0.81
58	1	0	0.78	0.22	0.27	0.73	0.32	0.68
59	1	0	0.81	0.19	0.22	0.78	0.27	0.73
60	1	0	0.77	0.23	0.22	0.78	0.29	0.71
**Classification accuracy**	**100%**		**100%**		**100%**		**100%**	
**Precision**	**1**		**1**		**1**		**1**	
**Recall**	**1**		**1**		**1**		**1**	
**F1 score**	**1**		**1**		**1**		**1**	

Supplementary Table 13. Summary of predicted probability for individual classes and overall classification accuracy of 4 classification models and predicted absolute values (chain length) for the regression model using peak parameters extracted from 60 SERS spectra from 60 10^{-5} M $GalCer_{16}$ blind test samples. Errors in classification are highlighted in red.

**$GalCer_{16}$**										
ML model	Classification model 1		Classification model 2		Classification model 3		Classification model 4		Regression model 5	
Sample no.	Loaded	Blank	Cerebroside	Monosaccharide	Saturated	Unsaturated	GlcCer	GalCer	Predicted chain length	Difference (%)
1	1	0	0.98	0.02	1	0	0.31	0.69	13.86	13.41
2	1	0	0.96	0.04	1	0	0.44	0.56	14.99	6.31
3	1	0	0.91	0.09	0.99	0.01	0.35	0.65	15.97	0.19
4	1	0	0.88	0.12	1	0	0.39	0.61	15.97	0.19
5	1	0	0.98	0.02	1	0	0.3	0.7	15.78	1.41
6	1	0	0.97	0.03	1	0	0.34	0.66	10.86	32.13
7	1	0	0.91	0.09	1	0	0.17	0.83	15.97	0.19
8	1	0	0.99	0.01	1	0	0.44	0.56	15.97	0.19
9	1	0	0.97	0.03	1	0	0.3	0.7	15.97	0.19
10	1	0	0.97	0.03	1	0	0.23	0.77	15.31	4.31
11	1	0	0.81	0.19	0.98	0.02	0.34	0.66	15.97	0.19
12	1	0	0.95	0.05	1	0	0.21	0.79	15.98	0.16
13	1	0	0.98	0.02	0.99	0.01	0.28	0.72	15.98	0.16
14	1	0	0.73	0.27	0.91	0.09	0.22	0.78	15.97	0.19
15	1	0	0.98	0.02	1	0	0.18	0.82	15.98	0.16
16	1	0	0.95	0.05	1	0	0.22	0.78	13.57	15.22
17	1	0	0.99	0.01	1	0	0.48	0.52	15.98	0.16
18	1	0	0.97	0.03	1	0	0.26	0.74	15.30	4.41
19	1	0	0.98	0.02	1	0	0.36	0.64	15.97	0.19
20	1	0	0.96	0.04	0.99	0.01	0.32	0.68	15.97	0.19
21	1	0	0.77	0.23	0.97	0.03	0.16	0.84	15.97	0.19
22	1	0	0.85	0.15	0.98	0.02	0.41	0.59	15.97	0.19
23	1	0	0.91	0.09	0.97	0.03	0.44	0.56	15.97	0.19
24	1	0	0.95	0.05	0.99	0.01	0.4	0.6	11.59	27.56
25	1	0	0.95	0.05	1	0	0.35	0.65	15.98	0.16
26	1	0	0.96	0.04	1	0	0.37	0.63	15.72	1.78
27	1	0	0.91	0.09	1	0	0.29	0.71	15.97	0.19
28	1	0	0.94	0.06	1	0	0.32	0.68	15.08	5.75
29	1	0	0.95	0.05	1	0	0.36	0.64	15.98	0.16
30	1	0	0.87	0.13	1	0	0.29	0.71	15.97	0.19
31	1	0	0.94	0.06	0.99	0.01	0.41	0.59	15.97	0.19
32	1	0	0.93	0.07	1	0	0.39	0.61	15.98	0.16
33	1	0	0.81	0.19	1	0	0.31	0.69	15.97	0.19
34	1	0	0.75	0.25	0.98	0.02	0.39	0.61	15.97	0.19
35	1	0	0.86	0.14	0.96	0.04	0.4	0.6	15.97	0.19
36	1	0	0.99	0.01	1	0	0.42	0.58	15.97	0.19
37	1	0	0.93	0.07	1	0	0.36	0.64	15.71	1.84
38	1	0	0.97	0.03	1	0	0.29	0.71	15.98	0.16
39	1	0	0.95	0.05	1	0	0.22	0.78	15.97	0.19
40	1	0	0.95	0.05	1	0	0.3	0.7	13.54	15.38
41	1	0	0.97	0.03	0.98	0.02	0.16	0.84	15.98	0.16
42	1	0	0.98	0.02	1	0	0.22	0.78	15.97	0.19
43	1	0	0.8	0.2	0.99	0.01	0.36	0.64	15.55	2.84
44	1	0	0.94	0.06	1	0	0.35	0.65	15.97	0.19
45	1	0	0.93	0.07	1	0	0.42	0.58	15.98	0.16
46	1	0	0.89	0.11	0.99	0.01	0.41	0.59	15.98	0.16

47	1	0	0.98	0.02	1	0	0.18	0.82	15.97	0.19
48	1	0	0.97	0.03	1	0	0.21	0.79	15.98	0.16
49	1	0	0.92	0.08	0.99	0.01	0.3	0.7	15.98	0.16
50	1	0	0.97	0.03	1	0	0.31	0.69	15.98	0.16
51	1	0	0.93	0.07	1	0	0.29	0.71	15.97	0.19
52	1	0	0.96	0.04	1	0	0.43	0.57	15.97	0.19
53	1	0	0.99	0.01	1	0	0.24	0.76	15.97	0.19
54	1	0	0.98	0.02	1	0	0.31	0.69	15.81	1.22
55	1	0	0.97	0.03	1	0	0.27	0.73	15.97	0.19
56	1	0	0.94	0.06	1	0	0.34	0.66	15.97	0.19
57	1	0	0.94	0.06	1	0	0.29	0.71	15.14	5.38
58	1	0	0.98	0.02	1	0	0.21	0.79	15.97	0.19
59	1	0	0.69	0.31	1	0	0.39	0.61	14.09	11.97
60	1	0	0.96	0.04	1	0	0.21	0.79	15.97	0.19
**Classification accuracy**	**100%**		**100%**		**100%**		**100%**			
**Precision**	**1**		**1**		**1**		**1**			
**Recall**	**1**		**1**		**1**		**1**			
**F1 score**	**1**		**1**		**1**		**1**			
**Mean**									15.58	2.65
**Std (+)**									1.00	6.27

Supplementary Table 14. Summary of predicted probability for individual classes and overall classification accuracy of 4 classification models and predicted absolute values (chain length) for the regression model using peak parameters extracted from 60 SERS spectra from 60 10^{-6} M $GalCer_{16}$ blind test samples. Errors in classification are highlighted in red.

**$GalCer_{16}$**										
ML model	Classification model 1		Classification model 2		Classification model 3		Classification model 4		Regression model 5	
Sample no.	Loaded	Blank	Cerebroside	Monosaccharide	Saturated	Unsaturated	GlcCer	GalCer	Predicted chain length	Difference (%)
1	1	0	0.85	0.15	1	0	0.33	0.67	17.00	6.27
2	1	0	0.65	0.35	0.99	0.01	0.23	0.77	16.05	0.34
3	1	0	0.85	0.15	0.99	0.01	0.41	0.59	16.15	0.97
4	1	0	0.81	0.19	1	0	0.17	0.83	16.12	0.78
5	1	0	0.76	0.24	0.9	0.1	0.19	0.81	16.05	0.34
6	1	0	0.89	0.11	0.99	0.01	0.25	0.75	15.90	0.64
7	1	0	0.91	0.09	0.99	0.01	0.3	0.7	16.08	0.50
8	1	0	0.84	0.16	1	0	0.43	0.57	16.05	0.34
9	1	0	0.85	0.15	0.99	0.01	0.47	0.53	16.05	0.34
10	1	0	0.79	0.21	0.99	0.01	0.24	0.76	16.14	0.87
11	1	0	0.7	0.3	0.99	0.01	0.38	0.62	16.09	0.56
12	1	0	0.93	0.07	1	0	0.34	0.66	16.07	0.47
13	1	0	0.68	0.32	0.92	0.08	0.35	0.65	16.28	1.72
14	1	0	0.86	0.14	0.9	0.1	0.44	0.56	16.05	0.34
15	1	0	0.91	0.09	0.99	0.01	0.38	0.62	16.14	0.91
16	1	0	0.89	0.11	1	0	0.39	0.61	16.23	1.41
17	1	0	0.87	0.13	1	0	0.23	0.77	16.15	0.97
18	1	0	0.86	0.14	0.99	0.01	0.59	0.41*	16.05	0.34
19	1	0	0.92	0.08	1	0	0.23	0.77	16.03	0.21
20	1	0	0.95	0.05	1	0	0.22	0.78	16.05	0.34
21	1	0	0.8	0.2	0.94	0.06	0.21	0.79	16.50	3.11
22	1	0	0.66	0.34	0.96	0.04	0.43	0.57	16.47	2.92
23	1	0	0.68	0.32	0.97	0.03	0.1	0.9	16.54	3.39
24	1	0	0.68	0.32	0.99	0.01	0.26	0.74	15.96	0.26
25	1	0	0.93	0.07	1	0	0.24	0.76	16.23	1.41
26	1	0	0.87	0.13	1	0	0.36	0.64	16.05	0.34
27	1	0	0.79	0.21	1	0	0.4	0.6	16.18	1.09
28	1	0	0.77	0.23	0.99	0.01	0.26	0.74	15.76	1.48
29	1	0	0.86	0.14	0.96	0.04	0.4	0.6	16.18	1.13
30	1	0	0.82	0.18	1	0	0.26	0.74	16.15	0.97
31	1	0	0.67	0.33	0.99	0.01	0.21	0.79	15.23	4.82
32	1	0	0.82	0.18	0.98	0.02	0.19	0.81	15.85	0.95
33	1	0	0.86	0.14	0.97	0.03	0.25	0.75	15.96	0.23
34	1	0	0.88	0.12	0.94	0.06	0.43	0.57	16.15	0.97
35	1	0	0.92	0.08	0.99	0.01	0.33	0.67	16.05	0.34
36	1	0	0.88	0.12	0.94	0.06	0.24	0.76	16.05	0.34
37	1	0	0.94	0.06	1	0	0.21	0.79	16.61	3.80
38	1	0	0.89	0.11	0.99	0.01	0.13	0.87	16.15	0.97
39	1	0	0.77	0.23	1	0	0.42	0.58	16.20	1.25
40	1	0	0.93	0.07	0.99	0.01	0.25	0.75	16.28	1.72
41	1	0	0.62	0.38	0.97	0.03	0.42	0.58	16.05	0.34
42	1	0	0.91	0.09	1	0	0.21	0.79	16.00	0.01
43	1	0	0.95	0.05	1	0	0.17	0.83	16.15	0.97
44	1	0	0.92	0.08	1	0	0.2	0.8	16.15	0.97
45	1	0	0.88	0.12	0.99	0.01	0.48	0.52	16.15	0.97
46	1	0	0.93	0.07	0.99	0.01	0.17	0.83	16.05	0.34

47	1	0	0.97	0.03	1	0	0.2	0.8	16.03	0.21
48	1	0	0.86	0.14	0.96	0.04	0.41	0.59	16.05	0.34
49	1	0	0.91	0.09	0.97	0.03	0.32	0.68	15.92	0.51
50	1	0	0.93	0.07	1	0	0.21	0.79	16.05	0.34
51	1	0	0.89	0.11	1	0	0.28	0.72	16.15	0.97
52	1	0	0.59	0.41	0.93	0.07	0.34	0.66	16.12	0.78
53	1	0	0.84	0.16	0.98	0.02	0.49	0.51	16.15	0.97
54	1	0	0.64	0.36	0.99	0.01	0.47	0.53	15.92	0.48
55	1	0	0.89	0.11	0.95	0.05	0.42	0.58	16.26	1.60
56	1	0	0.89	0.11	0.99	0.01	0.48	0.52	16.15	0.97
57	1	0	0.75	0.25	0.9	0.1	0.42	0.58	16.28	1.72
58	1	0	0.76	0.24	0.97	0.03	0.41	0.59	16.13	0.81
59	1	0	0.88	0.12	0.97	0.03	0.38	0.62	16.05	0.34
60	1	0	0.88	0.12	0.99	0.01	0.44	0.56	16.05	0.34
**Classification accuracy**	**100%**		**100%**		**100%**		**98%**			
**Precision**	**1**		**1**		**1**		**0.99**			
**Recall**	**1**		**1**		**1**		**0.99**			
**F1 score**	**1**		**1**		**1**		**0.99**			
**Mean**									16.12	1.07
**Std (±)**									0.22	1.15

Supplementary Table 15. Summary of predicted probability for individual classes and overall classification accuracy of 4 classification models and predicted absolute values (chain length) for the regression model using peak parameters extracted from 60 SERS spectra from 60 10^{-7} M $GalCer_{16}$ blind test samples. Errors in classification are highlighted in red.

**$GalCer_{16}$**										
ML model	Classification model 1		Classification model 2		Classification model 3		Classification model 4		Regression model 5	
Sample no.	Loaded	Blank	Cerebroside	Monosaccharide	Saturated	Unsaturated	GlcCer	GalCer	Predicted chain length	Difference (%)
1	1	0	0.04	0.96	0.94	0.06	0.36	0.64	16.16	1.00
2	1	0	0.03	0.97	1	0	0.47	0.53	16.32	2.00
3	1	0	0.06	0.94	0.92	0.08	0.39	0.61	16.40	2.50
4	1	0	0.04	0.96	0.98	0.02	0.43	0.57	16.33	2.06
5	1	0	0.02	0.98	1	0	0.28	0.72	16.26	1.63
6	1	0	0.02	0.98	1	0	0.38	0.62	16.24	1.50
7	1	0	0.03	0.97	1	0	0.45	0.55	16.29	1.81
8	1	0	0.05	0.95	1	0	0.55	0.45*	16.34	2.13
9	1	0	0.03	0.97	1	0	0.49	0.51	17.24	7.75
10	1	0	0.04	0.96	0.99	0.01	0.32	0.68	16.33	2.06
11	1	0	0.01	0.99	0.99	0.01	0.36	0.64	16.41	2.56
12	1	0	0.1	0.9	0.79	0.21	0.43	0.57	16.27	1.69
13	1	0	0.09	0.91	0.98	0.02	0.4	0.6	16.31	1.94
14	1	0	0.04	0.96	1	0	0.41	0.59	16.39	2.44
15	1	0	0.01	0.99	1	0	0.41	0.59	15.31	4.31
16	1	0	0.07	0.93	0.96	0.04	0.43	0.57	16.43	2.69
17	1	0	0.05	0.95	0.99	0.01	0.44	0.56	16.49	3.06
18	1	0	0.12	0.88	0.98	0.02	0.65	0.35*	16.37	2.31
19	1	0	0.02	0.98	1	0	0.42	0.58	16.25	1.56
20	1	0	0.06	0.94	1	0	0.51	0.49*	16.47	2.94
21	1	0	0.02	0.98	0.98	0.02	0.35	0.65	16.30	1.88
22	1	0	0.05	0.95	0.96	0.04	0.38	0.62	16.42	2.63
23	1	0	0.01	0.99	0.99	0.01	0.32	0.68	16.29	1.81
24	1	0	0.03	0.97	0.98	0.02	0.31	0.69	16.46	2.88
25	1	0	0.01	0.99	1	0	0.3	0.7	16.31	1.94
26	1	0	0.02	0.98	0.99	0.01	0.33	0.67	16.34	2.13
27	1	0	0.02	0.98	0.99	0.01	0.41	0.59	16.40	2.50
28	1	0	0.04	0.96	1	0	0.44	0.56	16.38	2.37
29	1	0	0.07	0.93	0.9	0.1	0.41	0.59	16.35	2.19
30	1	0	0.05	0.95	0.99	0.01	0.32	0.68	16.34	2.13
31	1	0	0.02	0.98	0.94	0.06	0.51	0.49*	16.20	1.25
32	1	0	0.04	0.96	0.99	0.01	0.4	0.6	16.24	1.50
33	1	0	0.02	0.98	0.99	0.01	0.31	0.69	18.27	14.19
34	1	0	0.03	0.97	0.98	0.02	0.38	0.62	16.32	2.00
35	1	0	0.05	0.95	0.97	0.03	0.39	0.61	16.32	2.00
36	1	0	0.09	0.91	0.93	0.07	0.48	0.52	16.25	1.56
37	1	0	0.06	0.94	0.96	0.04	0.32	0.68	16.31	1.94
38	1	0	0.05	0.95	1	0	0.47	0.53	16.33	2.06
39	1	0	0.09	0.91	0.98	0.02	0.38	0.62	16.21	1.31
40	1	0	0.03	0.97	0.99	0.01	0.33	0.67	16.24	1.50
41	1	0	0.05	0.95	0.98	0.02	0.35	0.65	16.35	2.19
42	1	0	0.08	0.92	0.92	0.08	0.43	0.57	16.28	1.75
43	1	0	0.08	0.92	0.98	0.02	0.45	0.55	16.38	2.37
44	1	0	0.01	0.99	0.99	0.01	0.31	0.69	16.33	2.06
45	1	0	0.1	0.9	0.94	0.06	0.34	0.66	16.25	1.56
46	1	0	0.1	0.9	0.94	0.06	0.38	0.62	16.32	2.00

47	1	0	0.1	0.9	0.97	0.03	0.41	0.59	16.20	1.25
48	1	0	0.07	0.93	0.93	0.07	0.43	0.57	16.31	1.94
49	1	0	0.04	0.96	0.99	0.01	0.36	0.64	16.32	2.00
50	1	0	0.09	0.91	0.91	0.09	0.33	0.67	16.32	2.00
51	1	0	0.05	0.95	0.99	0.01	0.4	0.6	16.36	2.25
52	1	0	0.07	0.93	0.99	0.01	0.38	0.62	16.42	2.63
53	1	0	0.07	0.93	0.99	0.01	0.44	0.56	16.29	1.81
54	1	0	0.06	0.94	0.92	0.08	0.35	0.65	14.35	10.31
55	1	0	0.1	0.9	0.95	0.05	0.4	0.6	14.32	10.50
56	1	0	0.06	0.94	1	0	0.46	0.54	16.34	2.13
57	1	0	0.08	0.92	0.88	0.12	0.41	0.59	16.23	1.44
58	1	0	0.06	0.94	0.87	0.13	0.39	0.61	14.32	10.50
59	1	0	0.12	0.88	0.89	0.11	0.45	0.55	16.25	1.56
60	1	0	0.09	0.91	0.87	0.13	0.38	0.62	16.33	2.06
**Classification accuracy**	**100%**		**100%**		**100%**		**93%**			
**Precision**	**1**		**1**		**1**		**0.93**			
**Recall**	**1**		**1**		**1**		**0.93**			
**F1 score**	**1**		**1**		**1**		**0.93**			
**Mean**									16.25	2.77
**Std (±)**									0.55	2.52

Supplementary Table 16. Summary of predicted probability for individual classes and overall classification accuracy of 4 classification models and predicted absolute values (chain length) for the regression model using peak parameters extracted from 60 SERS spectra from 60 10^{-8} M $GalCer_{16}$ blind test samples. Errors in classification are highlighted in red.

**$GalCer_{16}$**										
ML model	Classification model 1		Classification model 2		Classification model 3		Classification model 4		Regression model 5	
Sample no.	Loaded	Blank	Cerebroside	Monosaccharide	Saturated	Unsaturated	GlcCer	GalCer	Predicted chain length	Difference (%)
1	1	0	0.87	0.13	0.9	0.1	0.44	0.56	16.70	4.34
2	1	0	0.9	0.1	0.92	0.08	0.31	0.69	16.70	4.34
3	1	0	0.92	0.08	0.92	0.08	0.51	0.49*	16.91	5.66
4	1	0	0.93	0.07	0.92	0.08	0.34	0.66	16.84	5.26
5	1	0	0.93	0.07	0.92	0.08	0.45	0.55	16.70	4.34
6	1	0	0.93	0.07	0.93	0.07	0.28	0.72	16.37	2.31
7	1	0	0.93	0.07	0.94	0.06	0.32	0.68	16.75	4.67
8	1	0	0.93	0.07	0.94	0.06	0.32	0.68	16.70	4.34
9	1	0	0.93	0.07	0.94	0.06	0.63	0.37*	16.70	4.34
10	1	0	0.94	0.06	0.95	0.05	0.47	0.53	16.87	5.46
11	1	0	0.94	0.06	0.95	0.05	0.35	0.65	16.77	4.80
12	1	0	0.94	0.06	0.95	0.05	0.53	0.47*	16.74	4.61
13	1	0	0.95	0.05	0.95	0.05	0.36	0.64	17.16	7.23
14	1	0	0.95	0.05	0.95	0.05	0.32	0.68	16.70	4.34
15	1	0	0.95	0.05	0.95	0.05	0.3	0.7	16.88	5.53
16	1	0	0.95	0.05	0.96	0.04	0.33	0.67	17.05	6.58
17	1	0	0.95	0.05	0.96	0.04	0.44	0.56	16.91	5.66
18	1	0	0.95	0.05	0.96	0.04	0.37	0.63	16.70	4.34
19	1	0	0.95	0.05	0.96	0.04	0.31	0.69	16.65	4.08
20	1	0	0.96	0.04	0.96	0.04	0.31	0.69	16.70	4.34
21	1	0	0.96	0.04	0.96	0.04	0.39	0.61	17.62	10.12
22	1	0	0.96	0.04	0.96	0.04	0.44	0.56	17.56	9.73
23	1	0	0.96	0.04	0.97	0.03	0.37	0.63	17.71	10.71
24	1	0	0.96	0.04	0.97	0.03	0.31	0.69	16.50	3.10
25	1	0	0.96	0.04	0.97	0.03	0.35	0.65	17.05	6.58
26	1	0	0.96	0.04	0.97	0.03	0.43	0.57	16.70	4.34
27	1	0	0.96	0.04	0.97	0.03	0.33	0.67	16.95	5.92
28	1	0	0.96	0.04	0.97	0.03	0.28	0.72	16.09	0.54
29	1	0	0.96	0.04	0.97	0.03	0.25	0.75	16.96	5.98
30	1	0	0.97	0.03	0.97	0.03	0.21	0.79	16.91	5.66
31	1	0	0.97	0.03	0.97	0.03	0.24	0.76	14.97	6.42
32	1	0	0.97	0.03	0.97	0.03	0.59	0.41*	16.26	1.65
33	1	0	0.97	0.03	0.97	0.03	0.33	0.67	16.51	3.16
34	1	0	0.97	0.03	0.97	0.03	0.23	0.77	16.91	5.66
35	1	0	0.97	0.03	0.97	0.03	0.3	0.7	16.70	4.34
36	1	0	0.98	0.02	0.98	0.02	0.28	0.72	16.70	4.34
37	1	0	0.98	0.02	0.98	0.02	0.35	0.65	17.85	11.56
38	1	0	0.98	0.02	0.98	0.02	0.32	0.68	16.91	5.66
39	1	0	0.98	0.02	0.98	0.02	0.28	0.72	17.00	6.25
40	1	0	0.98	0.02	0.98	0.02	0.35	0.65	17.16	7.23
41	1	0	0.98	0.02	0.98	0.02	0.31	0.69	16.70	4.34
42	1	0	0.98	0.02	0.98	0.02	0.4	0.6	16.58	3.62
43	1	0	0.98	0.02	0.98	0.02	0.48	0.52	16.91	5.66
44	1	0	0.98	0.02	0.98	0.02	0.23	0.77	16.91	5.66
45	1	0	0.98	0.02	0.98	0.02	0.31	0.69	16.91	5.66
46	1	0	0.99	0.01	0.98	0.02	0.36	0.64	16.70	4.34

47	1	0	0.99	0.01	0.99	0.01	0.35	0.65	16.65	4.08
48	1	0	0.99	0.01	0.99	0.01	0.24	0.76	16.70	4.34
49	1	0	0.99	0.01	0.99	0.01	0.31	0.69	16.41	2.57
50	1	0	0.99	0.01	0.99	0.01	0.32	0.68	16.70	4.34
51	1	0	0.99	0.01	1	0	0.26	0.74	16.91	5.66
52	1	0	0.99	0.01	1	0	0.3	0.7	16.84	5.26
53	1	0	0.99	0.01	1	0	0.28	0.72	16.91	5.66
54	1	0	0.99	0.01	1	0	0.48	0.52	16.42	2.64
55	1	0	0.99	0.01	1	0	0.33	0.67	17.12	6.97
56	1	0	0.99	0.01	1	0	0.31	0.69	16.91	5.66
57	1	0	0.99	0.01	1	0	0.23	0.77	17.16	7.23
58	1	0	0.99	0.01	1	0	0.36	0.64	16.85	5.33
59	1	0	0.99	0.01	1	0	0.34	0.66	16.70	4.34
60	1	0	1	0	1	0	0.36	0.64	16.70	4.34
**Classification accuracy**	**100%**		**100%**		**100%**		**93%**			
**Precision**	**1**		**1**		**1**		**0.93**			
**Recall**	**1**		**1**		**1**		**0.93**			
**F1 score**	**1**		**1**		**1**		**0.93**			
**Mean**									16.80	5.22
**Std (+)**									0.39	1.93

Supplementary Table 17. Summary of predicted probability for individual classes and overall classification accuracy of 4 classification models and predicted absolute values (chain length) for the regression model using peak parameters extracted from 60 SERS spectra from 60 10^{-9} M $GalCer_{16}$ blind test samples. Errors in classification are highlighted in red.

$GalCer_{16}$										
ML model	Classification model 1		Classification model 2		Classification model 3		Classification model 4		Regression model 5	
Sample no.	Loaded	Blank	Cerebroside	Monosaccharide	Saturated	Unsaturated	GlcCer	GalCer	Predicted chain length	Difference (%)
1	1	0	0.98	0.02	0.97	0.03	0.41	0.59	16.66	4.10
2	1	0	0.96	0.04	0.92	0.08	0.34	0.66	15.54	2.85
3	1	0	0.98	0.02	0.97	0.03	0.35	0.65	15.61	2.45
4	1	0	0.99	0.01	1	0	0.37	0.63	17.55	9.68
5	1	0	0.97	0.03	0.97	0.03	0.5	0.5	15.61	2.45
6	1	0	0.94	0.06	0.9	0.1	0.37	0.63	17.61	10.08
7	1	0	0.98	0.02	0.96	0.04	0.32	0.68	17.61	10.08
8	1	0	0.98	0.02	0.98	0.02	0.25	0.75	15.61	2.45
9	1	0	0.95	0.05	0.98	0.02	0.42	0.58	16.72	4.52
10	1	0	0.97	0.03	1	0	0.26	0.74	15.61	2.45
11	1	0	0.98	0.02	0.96	0.04	0.31	0.69	15.61	2.45
12	1	0	0.98	0.02	0.98	0.02	0.31	0.69	15.61	2.45
13	1	0	0.98	0.02	0.99	0.01	0.43	0.57	18.81	17.59
14	1	0	0.98	0.02	0.99	0.01	0.23	0.77	15.54	2.85
15	1	0	0.97	0.03	0.98	0.02	0.35	0.65	15.06	5.87
16	1	0	0.94	0.06	1	0	0.6	0.4*	17.61	10.08
17	1	0	0.95	0.05	0.96	0.04	0.42	0.58	15.61	2.45
18	1	0	0.94	0.06	1	0	0.54	0.46*	15.61	2.45
19	1	0	0.97	0.03	0.99	0.01	0.34	0.66	18.82	17.60
20	1	0	0.94	0.06	1	0	0.43	0.57	15.61	2.45
21	1	0	0.99	0.01	0.93	0.07	0.43	0.57	15.55	2.80
22	1	0	0.97	0.03	1	0	0.42	0.58	15.54	2.85
23	1	0	0.99	0.01	0.97	0.03	0.34	0.66	15.61	2.45
24	1	0	0.97	0.03	0.99	0.01	0.24	0.76	15.54	2.85
25	1	0	0.98	0.02	0.93	0.07	0.24	0.76	15.64	2.22
26	1	0	0.97	0.03	1	0	0.27	0.73	15.61	2.45
27	1	0	0.98	0.02	0.92	0.08	0.47	0.53	18.69	16.82
28	1	0	0.99	0.01	0.99	0.01	0.29	0.71	15.61	2.45
29	1	0	0.97	0.03	0.92	0.08	0.37	0.63	14.05	12.19
30	1	0	0.98	0.02	0.99	0.01	0.35	0.65	15.61	2.45
31	1	0	0.98	0.02	1	0	0.32	0.68	15.61	2.45
32	1	0	0.98	0.02	0.99	0.01	0.23	0.77	17.61	10.08
33	1	0	0.98	0.02	0.98	0.02	0.36	0.64	17.61	10.05
34	1	0	0.97	0.03	0.98	0.02	0.35	0.65	15.56	2.76
35	1	0	0.97	0.03	0.97	0.03	0.36	0.64	15.54	2.85
36	1	0	0.95	0.05	1	0	0.46	0.54	15.54	2.85
37	1	0	0.99	0.01	0.96	0.04	0.29	0.71	15.61	2.45
38	1	0	0.99	0.01	0.97	0.03	0.38	0.62	15.61	2.45
39	1	0	0.99	0.01	1	0	0.42	0.58	15.61	2.45
40	1	0	0.99	0.01	0.97	0.03	0.29	0.71	18.81	17.59
41	1	0	0.98	0.02	0.97	0.03	0.43	0.57	15.54	2.85
42	1	0	0.98	0.02	0.9	0.1	0.27	0.73	16.66	4.13
43	1	0	0.95	0.05	0.99	0.01	0.41	0.59	15.61	2.45
44	1	0	0.96	0.04	0.9	0.1	0.38	0.62	18.61	16.30
45	1	0	0.98	0.02	1	0	0.28	0.72	15.61	2.45
46	1	0	0.92	0.08	0.92	0.08	0.36	0.64	16.90	5.62

47	1	0	0.98	0.02	0.97	0.03	0.32	0.68	15.54	2.85
48	1	0	0.94	0.06	1	0	0.37	0.63	17.61	10.05
49	1	0	0.99	0.01	0.98	0.02	0.36	0.64	15.54	2.85
50	1	0	0.9	0.1	0.9	0.1	0.5	0.5	17.61	10.05
51	1	0	0.97	0.03	0.95	0.05	0.41	0.59	17.61	10.05
52	1	0	0.99	0.01	0.95	0.05	0.28	0.72	13.71	14.33
53	1	0	0.94	0.06	0.96	0.04	0.3	0.7	15.54	2.85
54	1	0	0.99	0.01	0.99	0.01	0.35	0.65	15.61	2.45
55	1	0	0.98	0.02	0.98	0.02	0.37	0.63	14.61	8.70
56	1	0	0.98	0.02	0.96	0.04	0.35	0.65	15.61	2.45
57	1	0	0.98	0.02	0.98	0.02	0.37	0.63	16.53	3.34
58	1	0	0.96	0.04	0.95	0.05	0.44	0.56	17.55	9.68
59	1	0	0.97	0.03	1	0	0.37	0.63	15.61	2.45
60	1	0	0.99	0.01	0.9	0.1	0.32	0.68	15.61	2.45
**Classification accuracy**	**100%**		**100%**		**100%**		**97%**			
**Precision**	**1**		**1**		**1**		**0.97**			
**Recall**	**1**		**1**		**1**		**0.97**			
**F1 score**	**1**		**1**		**1**		**0.97**			
**Mean**									16.20	5.69
**Std (±)**									1.18	4.76

Supplementary Table 18. Summary of predicted probability for individual classes and overall classification accuracy of 4 classification models and predicted absolute values (chain length) for the regression model using peak parameters extracted from 60 SERS spectra from 60 10^{-10} M $GalCer_{16}$ blind test samples. Errors in classification are highlighted in red.

**$GalCer_{16}$**										
ML model	Classification model 1		Classification model 2		Classification model 3		Classification model 4		Regression model 5	
Sample no.	Loaded	Blank	Cerebroside	Monosaccharide	Saturated	Unsaturated	GlcCer	GalCer	Predicted chain length	Difference (%)
1	1	0	0.99	0.01	0.97	0.03	0.37	0.63	15.62	2.39
2	1	0	0.95	0.05	0.97	0.03	0.46	0.54	14.51	9.32
3	1	0	0.98	0.02	0.97	0.03	0.47	0.53	14.57	8.93
4	1	0	0.98	0.02	0.97	0.03	0.43	0.57	16.51	3.18
5	1	0	0.96	0.04	0.97	0.03	0.44	0.56	14.57	8.93
6	1	0	0.98	0.02	0.97	0.03	0.52	0.48*	16.57	3.57
7	1	0	0.91	0.09	0.92	0.08	0.4	0.6	16.57	3.57
8	1	0	0.96	0.04	0.96	0.04	0.45	0.55	14.57	8.93
9	1	0	0.97	0.03	0.97	0.03	0.47	0.53	15.69	1.97
10	1	0	0.94	0.06	0.97	0.03	0.43	0.57	14.57	8.93
11	1	0	0.97	0.03	0.99	0.01	0.4	0.6	14.57	8.93
12	1	0	0.96	0.04	0.96	0.04	0.41	0.59	14.57	8.93
13	1	0	0.96	0.04	0.95	0.05	0.43	0.57	17.77	11.07
14	1	0	0.98	0.02	1	0	0.48	0.52	14.51	9.32
15	1	0	0.96	0.04	0.97	0.03	0.44	0.56	14.03	12.34
16	1	0	0.91	0.09	0.99	0.01	0.29	0.71	16.57	3.57
17	1	0	0.91	0.09	1	0	0.29	0.71	14.57	8.93
18	1	0	0.97	0.03	0.99	0.01	0.49	0.51	14.57	8.93
19	1	0	0.95	0.05	0.94	0.06	0.47	0.53	17.77	11.08
20	1	0	0.97	0.03	0.99	0.01	0.39	0.61	14.57	8.93
21	1	0	0.97	0.03	1	0	0.34	0.66	14.52	9.28
22	1	0	0.98	0.02	1	0	0.43	0.57	14.51	9.32
23	1	0	0.98	0.02	0.96	0.04	0.46	0.54	14.57	8.93
24	1	0	0.94	0.06	0.96	0.04	0.61	0.39*	14.51	9.32
25	1	0	0.98	0.02	1	0	0.41	0.59	14.51	9.32
26	1	0	0.95	0.05	0.89	0.11	0.45	0.55	14.57	8.93
27	1	0	0.95	0.05	0.98	0.02	0.29	0.71	17.65	10.31
28	1	0	0.96	0.04	0.97	0.03	0.47	0.53	14.57	8.93
29	1	0	0.95	0.05	1	0	0.45	0.55	13.02	18.64
30	1	0	0.93	0.07	0.98	0.02	0.4	0.6	14.57	8.93
31	1	0	0.98	0.02	1	0	0.29	0.71	14.57	8.93
32	1	0	0.99	0.01	0.98	0.02	0.35	0.65	16.57	3.57
33	1	0	0.95	0.05	0.98	0.02	0.48	0.52	14.57	8.93
34	1	0	0.93	0.07	0.93	0.07	0.52	0.48*	14.52	9.23
35	1	0	0.95	0.05	0.98	0.02	0.47	0.53	14.51	9.32
36	1	0	0.9	0.1	0.97	0.03	0.43	0.57	14.51	9.32
37	1	0	0.95	0.05	1	0	0.37	0.63	14.57	8.93
38	1	0	0.94	0.06	0.99	0.01	0.43	0.57	14.57	8.93
39	1	0	0.94	0.06	0.95	0.05	0.51	0.49*	14.57	8.93
40	1	0	0.93	0.07	0.97	0.03	0.52	0.48*	17.77	11.07
41	1	0	0.96	0.04	0.98	0.02	0.55	0.45*	14.51	9.32
42	1	0	0.99	0.01	1	0	0.57	0.43*	14.03	12.34
43	1	0	0.95	0.05	0.94	0.06	0.45	0.55	14.57	8.93
44	1	0	0.96	0.04	0.99	0.01	0.43	0.57	14.57	8.93
45	1	0	0.97	0.03	0.99	0.01	0.38	0.62	14.57	8.93
46	1	0	0.95	0.05	0.99	0.01	0.47	0.53	15.86	0.88

47	1	0	0.97	0.03	0.99	0.01	0.45	0.55	14.51	9.32
48	1	0	0.94	0.06	0.97	0.03	0.45	0.55	14.57	8.93
49	1	0	0.94	0.06	0.91	0.09	0.45	0.55	14.51	9.32
50	1	0	0.95	0.05	0.93	0.07	0.48	0.52	14.57	8.93
51	1	0	0.95	0.05	0.93	0.07	0.47	0.53	14.57	8.93
52	1	0	0.89	0.11	0.99	0.01	0.45	0.55	12.68	20.78
53	1	0	0.91	0.09	0.97	0.03	0.44	0.56	14.51	9.32
54	1	0	0.95	0.05	1	0	0.41	0.59	14.57	8.93
55	1	0	0.94	0.06	0.98	0.02	0.52	0.48*	14.57	8.93
56	1	0	0.95	0.05	0.98	0.02	0.49	0.51	14.57	8.93
57	1	0	0.93	0.07	0.98	0.02	0.48	0.52	15.50	3.15
58	1	0	0.92	0.08	0.95	0.05	0.48	0.52	16.51	3.18
59	1	0	0.93	0.07	0.99	0.01	0.43	0.57	14.57	8.93
60	1	0	0.92	0.08	0.97	0.03	0.44	0.56	14.57	8.93
**Classification accuracy**	**100%**		**100%**		**100%**		**87%**			
**Precision**	**1**		**1**		**1**		**0.87**			
**Recall**	**1**		**1**		**1**		**0.87**			
**F1 score**	**1**		**1**		**1**		**0.87**			
**Mean**									14.96	8.61
**Std (+)**									1.06	3.29

Supplementary Table 19. Multiplex quantification blind test 1. Summary of experimental vs. predicted concentration (% composition) for 10 unknown samples of $GlcCer_{24:1}$ in a mixture containing both $GlcCer_{24:1}$ and $GalCer_{24:1}$ with a total concentration of 100 μM.

**Multiplex Quantification Blind Test 1**						
	$GlcCer_{24:1}$			$GalCer_{24:1}$		
Unknown sample no.	Actual concentration (%)	Predicted concentration (%)	Difference (%)	Actual concentration (%)	Predicted concentration (%)	Difference (%)
1	10	11.69	16.86	90	88.31	1.87
2	10	13.34	33.39	90	86.66	3.71
3	10	14.33	43.31	90	85.67	4.81
4	10	19.35	93.47	90	80.65	10.39
5	10	17.57	75.72	90	82.43	8.41
6	10	13.34	33.39	90	86.66	3.71
7	10	15.34	53.41	90	84.66	5.93
8	10	12.34	23.42	90	87.66	2.60
9	10	13.68	36.83	90	86.32	4.09
10	10	11.30	13.04	90	88.70	1.45
**Mean**		**14.23**	**42.28**		**85.77**	**4.70**
**Std (+)**		**2.43**	**24.31**		**2.43**	**2.70**

Supplementary Table 20. Multiplex quantification blind test 2. Summary of experimental vs. predicted concentration (% composition) for 10 unknown samples of $GlcCer_{24:1}$ in a mixture containing both $GlcCer_{24:1}$ and $GalCer_{24:1}$ with a total concentration of 100 μM.

**Multiplex Quantification Blind Test 2**						
	GlcCer24:1			GalCer24:1		
Unknown sample no.	Actual concentration (%)	Predicted concentration (%)	Difference (%)	Actual concentration (%)	Predicted concentration (%)	Difference (%)
1	40	42.38	5.94	60	57.62	3.97
2	40	39.54	1.14	60	60.46	0.77
3	40	40.27	0.69	60	59.73	0.45
4	40	36.40	9.00	60	63.60	6.00
5	40	40.10	0.25	60	59.90	0.17
6	40	33.61	15.97	60	66.39	10.65
7	40	36.47	8.83	60	63.53	5.88
8	40	42.46	6.15	60	57.54	4.10
9	40	36.68	8.29	60	63.32	5.53
10	40	38.68	3.31	60	61.32	2.20
**Mean**		**38.66**	**5.96**		**61.34**	**3.97**
**Std (+)**		**2.70**	**4.62**		**2.70**	**2.94**

Supplementary Table 21. Multiplex quantification blind test 3. Summary of experimental vs. predicted concentration (% composition) for 10 unknown samples of $GlcCer_{24:1}$ in a mixture containing both $GlcCer_{24:1}$ and $GalCer_{24:1}$ with a total concentration of 100 μM.

**Multiplex Quantification Blind Test 3**						
	$GlcCer_{24:1}$			$GalCer_{24:1}$		
Unknown sample no.	Actual concentration (%)	Predicted concentration (%)	Difference (%)	Actual concentration (%)	Predicted concentration (%)	Difference (%)
1	60	61.20	2.00	40	38.80	3.00
2	60	56.42	5.97	40	43.58	8.95
3	60	51.15	14.74	40	48.85	22.11
4	60	56.77	5.38	40	43.23	8.07
5	60	58.66	2.24	40	41.34	3.36
6	60	60.00	0.00	40	40.00	0.01
7	60	60.58	0.97	40	39.42	1.45
8	60	58.63	2.29	40	41.37	3.43
9	60	59.31	1.15	40	40.69	1.73
10	60	58.75	2.08	40	41.25	3.12
**Mean**		**58.15**	**3.68**		**41.85**	**5.52**
**Std (+)**		**2.73**	**4.09**		**2.73**	**6.13**

Supplementary Table 22. Pure analyte identification and quantification blind test 1. Summary of experimental vs. predicted class and concentration for 10 blind samples of $GalCer_{12}$ at 10^{-8} M. Errors in classification are highlighted in red.

Blind test samples of $GalCer_{12}$ at 10^{-8} M												
ML model	Classification model 1		Classification model 2		Classification model 3		Classification model 4		Regression model 5		Quantification model ($Galcer_{12}$)	
Sample no.	Loaded	Blank	Cerebroside	Monosaccharide	Saturated	Unsaturated	GlcCer	GalCer	Predicted chain length	Difference (%)	Predicted concentration (nM)	Difference (%)
1	1	0	0.7	0.3	0.51	0.49	0.26	0.74	12.16	1.33	12	20
2	1	0	0.7	0.3	0.61	0.39	0.35	0.65	12.51	4.25	10.01	0.1
3	1	0	0.77	0.23	0.61	0.39	0.26	0.74	12.45	3.75	9.4	6
4	1	0	0.8	0.2	0.48*	0.52	0.38	0.62	12.51	4.25	9.5	5
5	1	0	0.8	0.2	0.6	0.4	0.33	0.67	12.49	4.08	10.2	2
6	1	0	0.76	0.24	0.6	0.4	0.26	0.74	12.44	3.67	10.05	0.5
7	1	0	0.7	0.3	0.52	0.48	0.38	0.62	12.48	4.00	11	10
8	1	0	0.77	0.23	0.6	0.4	0.34	0.66	12.49	4.08	11.2	12
9	1	0	0.8	0.2	0.61	0.39	0.26	0.74	13.84	15.33	11.5	15
10	1	0	0.7	0.3	0.51	0.49	0.26	0.74	12.41	3.42	12	20
**Classification accuracy**	**100%**		**100%**		**90%**		**100%**					
**Precision**	**1**		**1**		**0.9**		**1**					
**Recall**	**1**		**1**		**0.9**		**1**					
**F1 score**	**1**		**1**		**0.9**		**1**					
**Mean**									**12.57**	**4.82**	**10.69**	**9.06**
**Std (±)**									**0.45**	**3.79**	**0.98**	**7.55**

Supplementary Table 23. Pure analyte identification and quantification blind test 1. Summary of experimental vs. predicted class and concentration for 10 blind samples of $GlcCer_{16}$ at 10^{-6} M. Errors in classification are highlighted in red.

**Blind test samples of $GlcCer_{16}$ at 10^{-6} M**												
ML model	Classification model 1		Classification model 2		Classification model 3		Classification model 4		Regression model 5		Quantification model ($Galcer_{12}$)	
Sample no.	Loaded	Blank	Cerebroside	Monosaccharide	Saturated	Unsaturated	GlcCer	GalCer	Predicted chain length	Difference (%)	Predicted concentration (uM)	Difference (%)
1	1	0	0.98	0.02	1	0	0.57	0.43	15.4	3.75	1.09	9
2	1	0	0.97	0.03	1	0	0.55	0.45	15.58	2.63	1.13	13
3	1	0	0.98	0.02	1	0	0.77	0.23	15.74	1.63	1.2	20
4	1	0	0.99	0.01	1	0	0.69	0.31	15.6	2.50	1.05	5
5	1	0	0.99	0.01	0.99	0.01	0.74	0.26	15.4	3.75	1.06	6
6	1	0	0.95	0.05	1	0	0.55	0.45	15.36	4.00	1.18	18
7	1	0	0.99	0.01	1	0	0.63	0.37	15.4	3.75	1.03	3
8	1	0	0.97	0.03	1	0	0.69	0.31	16.28	1.75	1.12	12
9	1	0	0.99	0.01	1	0	0.81	0.19	16.22	1.38	1.07	7
10	1	0	0.99	0.01	1	0	0.72	0.28	16.37	2.31	1.04	4
**Classification accuracy**	**100%**		**100%**		**100%**		**100%**					
**Precision**	**1**		**1**		**1**		**1**					
**Recall**	**1**		**1**		**1**		**1**					
**F1 score**	**1**		**1**		**1**		**1**					
**Mean**									**15.73**	**2.74**	**1.10**	**9.70**
**Std (±)**									**0.40**	**0.99**	**0.06**	**5.89**

Supplementary Table 24. Pure analyte identification and quantification blind test 1. Summary of experimental vs. predicted concentration for 10 blind samples of $GalCer_{24}$. Errors in classification are highlighted in red.

**Blind test samples of $GalCer_{24}$ at 10^{-9} M**												
ML model	Classification model 1		Classification model 2		Classification model 3		Classification model 4		Regression model 5		Quantification model ($Galcer_{12}$)	
Sample no.	Loaded	Blank	Cerebroside	Monosaccharide	Saturated	Unsaturated	GlcCer	GalCer	Predicted chain length	Difference (%)	Predicted concentration (nM)	Difference (%)
1	1	0	0.99	0.01	1	0	0.45	0.55	24.46	1.90	1.018	1.8
2	1	0	0.99	0.01	1	0	0.42	0.58	24.16	0.65	1.058	5.8
3	1	0	0.98	0.02	1	0	0.36	0.64	24.45	1.88	1.098	9.8
4	1	0	0.98	0.02	1	0	0.49	0.51	23.86	0.60	1.268	26.8
5	1	0	0.99	0.01	0.99	0.01	0.52	0.48*	24.46	1.90	1.028	2.8
6	1	0	0.92	0.08	0.97	0.03	0.37	0.63	24.67	2.79	1.148	14.8
7	1	0	0.99	0.01	1	0	0.55	0.45*	24.16	0.65	0.898	10.2
8	1	0	0.98	0.02	1	0	0.39	0.61	24.46	1.90	1.088	8.8
9	1	0	0.99	0.01	1	0	0.47	0.53	24.06	0.24	0.98038	1.962
10	1	0	0.99	0.01	0.96	0.04	0.4	0.6	24.18	0.74	1.008	0.8
**Classification accuracy**	**100%**		**100%**		**100%**		**80%**					
**Precision**	**1**		**1**		**1**		**0.8**					
**Recall**	**1**		**1**		**1**		**0.8**					
**F1 score**	**1**		**1**		**1**		**0.8**					
**Mean**									**24.29**	**1.33**	**1.06**	**8.36**
**Std (±)**									**0.25**	**0.84**	**0.10**	**7.93**

Supplementary references

1. Tao, A.; Sinsermsuksakul, P.; Yang, P., Polyhedral silver nanocrystals with distinct scattering signatures. *Angew. Chem. Int. Ed.* **2006,** *45* (28), 4597-4601.

2. Zhang, Z. M.; Chen, S.; Liang, Y. Z., Baseline correction using adaptive iteratively reweighted penalized least squares. *Analyst* **2010,** *135* (5), 1138-46.

3. Demšar, J.; Curk, T.; Erjavec, A.; Gorup, Č.; Hočevar, T.; Milutinovič, M.; Možina, M.; Polajnar, M.; Toplak, M.; Starič, A.; Štajdohar, M.; Umek, L.; Žagar, L.; Žbontar, J.; Žitnik, M.; Zupan, B., Orange: data mining toolbox in python. *J. Mach. Learn. Res.* **2013,** *14* (1), 2349–2353.
